# Supplementary material for: Activated glucocorticoid receptor is an estrogen receptor silencer in ER+ metastatic breast cancer
Source: EMBO Mol Med. 2025 Nov 19;18(1):151–86. doi: 10.1038/s44321-025-00342-z (PMC12808765; doi:10.1038/s44321-025-00342-z)
Supplement: Supplementary file 1 — Appendix [file 44321_2025_342_MOESM1_ESM.pdf]

Activated Glucocorticoid Receptor is an Estrogen Receptor Silencer in ER+ metastatic breast cancer

Appendix Figures

Table of Content

Appendix Figure S1: GR activation enhances reduces liver metastases in *ESR1* mutant models in presence of Paclitaxel. **(page 2)**

Appendix Figure S2: GR activation reduces the viability of cancer cells *in vitro.* **(page 4)**

Appendix Figure S3: Functional annotation of proteomics and RNA-Seq datasets. **(page 6)**

Appendix Figure S4: Cox regression multivariate analyses using GR activity signature. **(page 8)**

Appendix Figure S5: Immunohistochemistry of ER and GR proteins in ER+ patient samples. **(page 10)**

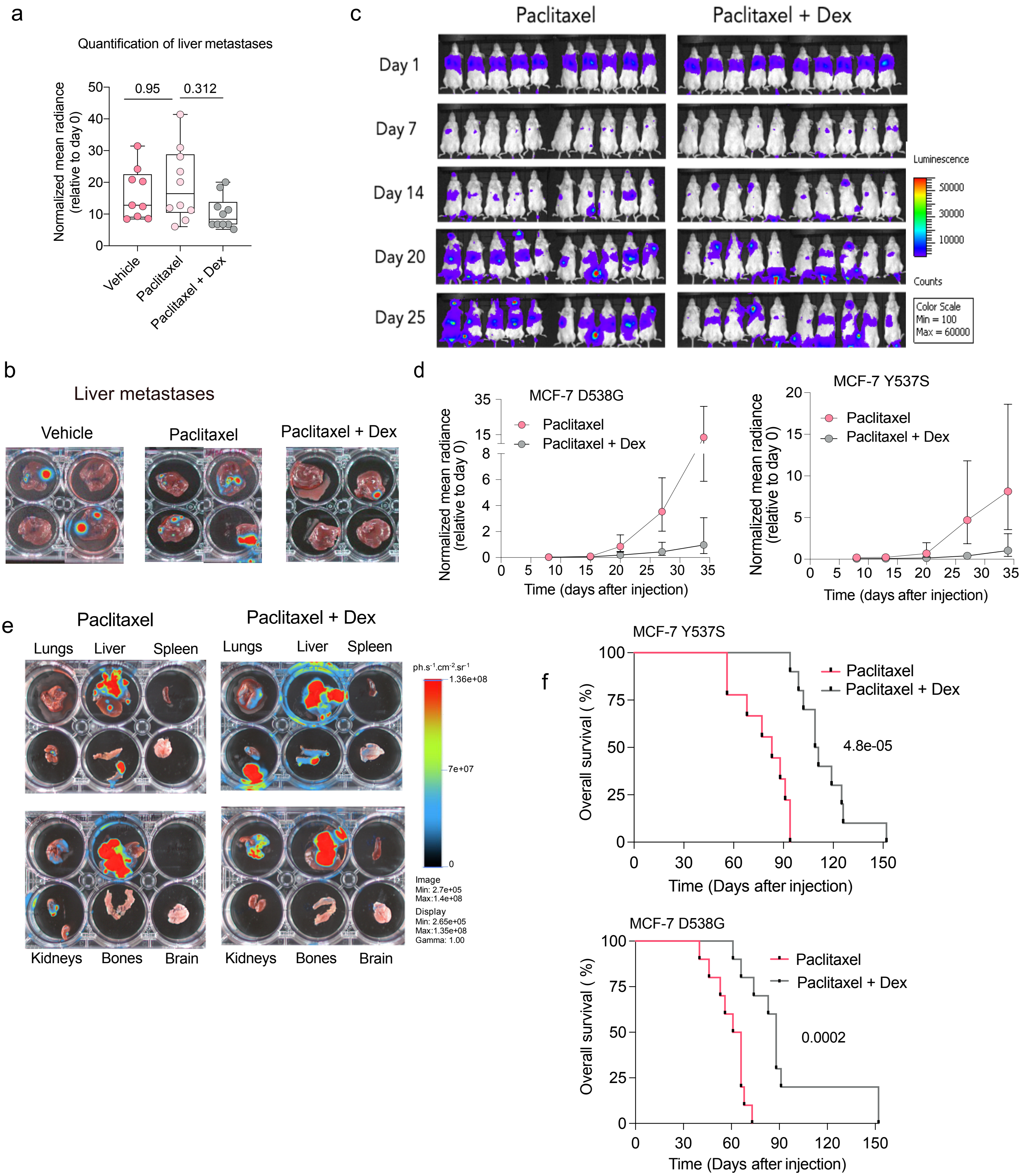

a
Quantification of liver metastases
0.95
0.312
Normalized mean radiance (relative to day 0)
Vehicle
Paclitaxel
Paclitaxel + Dex
c
Paclitaxel
Paclitaxel + Dex
Day 1
Day 7
Day 14
Day 20
Day 25
Luminescence
Counts
Color Scale Min = 100 Max = 60000
b
Liver metastases
Vehicle
Paclitaxel
Paclitaxel + Dex
d
MCF-7 D538G
MCF-7 Y537S
Paclitaxel
Paclitaxel + Dex
Normalized mean radiance (relative to day 0)
Time (days after injection)
e
Paclitaxel
Paclitaxel + Dex
Lungs
Liver
Spleen
Kidneys
Bones
Brain
1.36e+08
7e+07
Image Min: 2.7e+05 Max:1.4e+08
Display Min: 2.65e+05 Max:1.35e+08 Gamma: 1.00
f
MCF-7 Y537S
Overall survival (%)
Time (Days after injection)
4.8e-05
MCF-7 D538G
0.0002

Appendix Figure S1: GR activation reduces liver metastases in *ESR1* mutant models in presence of Paclitaxel.

a, Box plot showing the quantification of liver metastases at day 36; *n* = 9 - 10 mice per group. ns, non-significant, *$P < 0.05$; One-way ANOVA Kruskal-Wallis test. Boxes define the upper and lower quartiles; the central band indicates the median; whiskers define max. to min. values. **b,** Representative bioluminescence images of livers harvested upon sacrifice at day 36; *n* = 9 - 10 mice per group. Imaging of livers was performed with Newton Vilber. **c,** Bioluminescence imaging of mice intravenously injected with MCF-7 D538G cells treated with Paclitaxel or Paclitaxel in combination with Dex; *n* = 10 mice per group. Imaging was performed with IVIS. **d,** Graphs depicting the estimation of whole-body metastatic burden over time in mice intravenously injected with GR-activated or control MCF-7 D538G and Y537S cells treated with Paclitaxel alone or in combination with Dex. **e,** Representative bioluminescence images of distant organs collected upon manifestation of signs of distress; *n* = 10 mice per group. Imaging was performed with Newton Vilber. **f,** Kaplan Meier survival analyses of mice intravenously injected with MCF-7 D538G and Y537S cells treated with Paclitaxel or Paclitaxel in combination with Dex; *n* = 10 mice per group. Log-rank (Mantel-Cox) test.

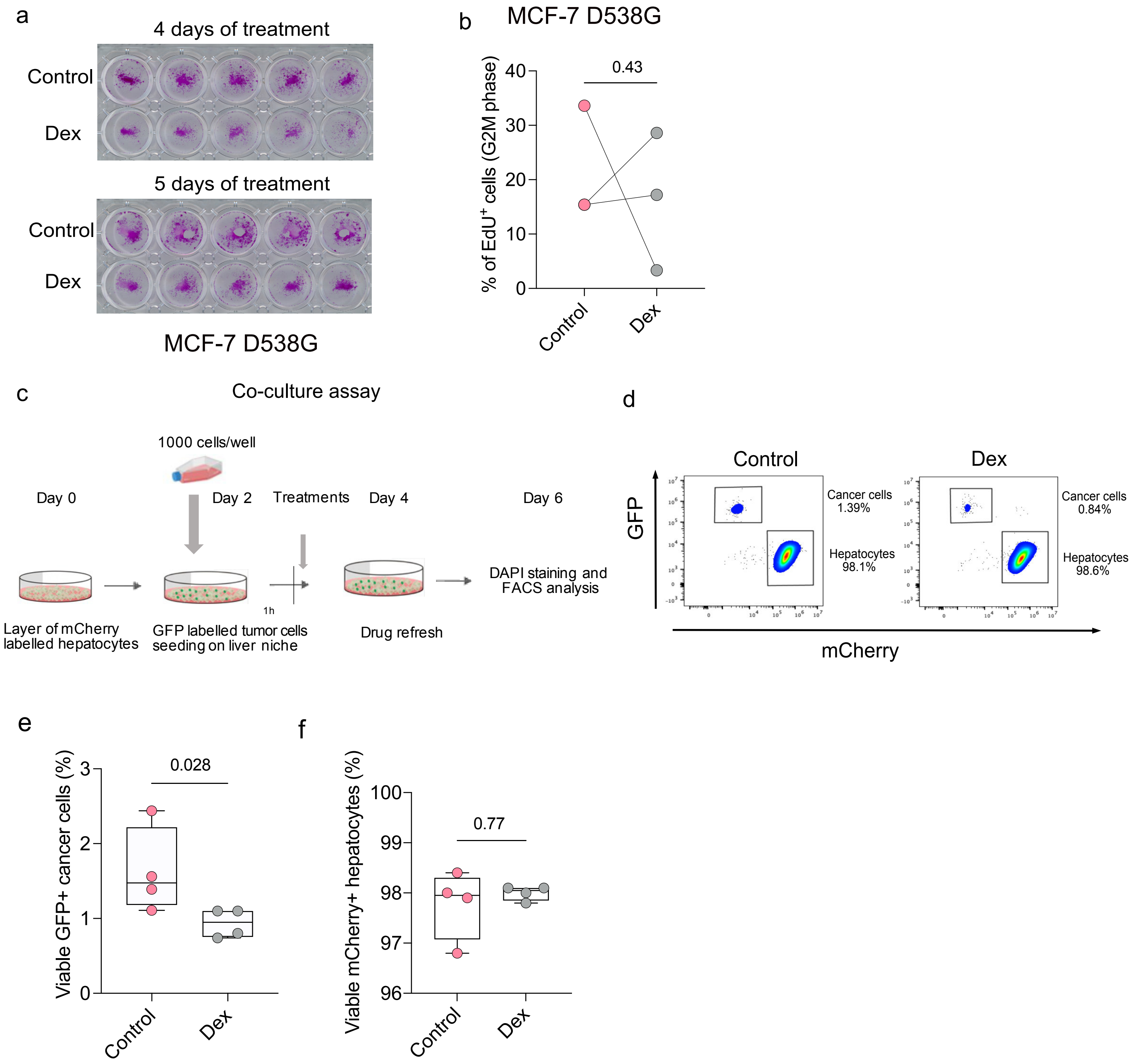
a
4 days of treatment
Control
Dex
5 days of treatment
Control
Dex
MCF-7 D538G
b
MCF-7 D538G
% of EdU+ cells (G2M phase)
0.43
40
30
20
10
0
Control
Dex
c
Co-culture assay
1000 cells/well
Day 0
Day 2
Treatments
Day 4
Day 6
1h
DAPI staining and FACS analysis
Layer of mCherry labelled hepatocytes
GFP labelled tumor cells seeding on liver niche
Drug refresh
d
Control
Dex
GFP
Cancer cells 1.39%
Hepatocytes 98.1%
Cancer cells 0.84%
Hepatocytes 98.6%
mCherry
e
Viable GFP+ cancer cells (%)
0.028
3
2
1
0
Control
Dex
f
Viable mCherry+ hepatocytes (%)
0.77
100
99
98
97
96
Control
Dex

Appendix Figure S2: GR activation reduces the viability of cancer cells *in vitro.*

a, Representative images of SRB assay plates after Dex treatment of MCF-7 D538G cells for 4 and 5 days; *n* = 3 biological replicates with 4 - 5 technical replicates each. **b,** Graph showing the percentage of EdU-stained live cells in G2/M phase as analyzed by FACS; *n* = 3 biological replicates. ns, non-significant; Two-tailed paired t-test. Individual values and lines represent pairing of data points. **c,** Experimental design of the co-culture of MCF-7 D538G cells in the liver niche. **d,** Representative flow cytometry dot plots of mCherry-labeled hepatocytes co-cultured with GFP-labelled MCF-7 D538G cells after treatment for 4 days with 700 nM Dex or vehicle. **e,** Quantification of GFP+ live cancer cells in a liver-like milieu; *n* = 4 independent experiments. *$P < 0.05$; Two-tailed Mann-Whitney test. Boxes define the upper and lower quartiles; a central band indicates the median; whiskers define max. to min. values. **f,** Quantification of mCherry+ hepatocytes after treatment; *n* = 4 independent experiments. ns, non-significant. Two-tailed Mann-Whitney test. Boxes define the upper and lower quartiles; a central band indicates the median; whiskers define max. to min. values.

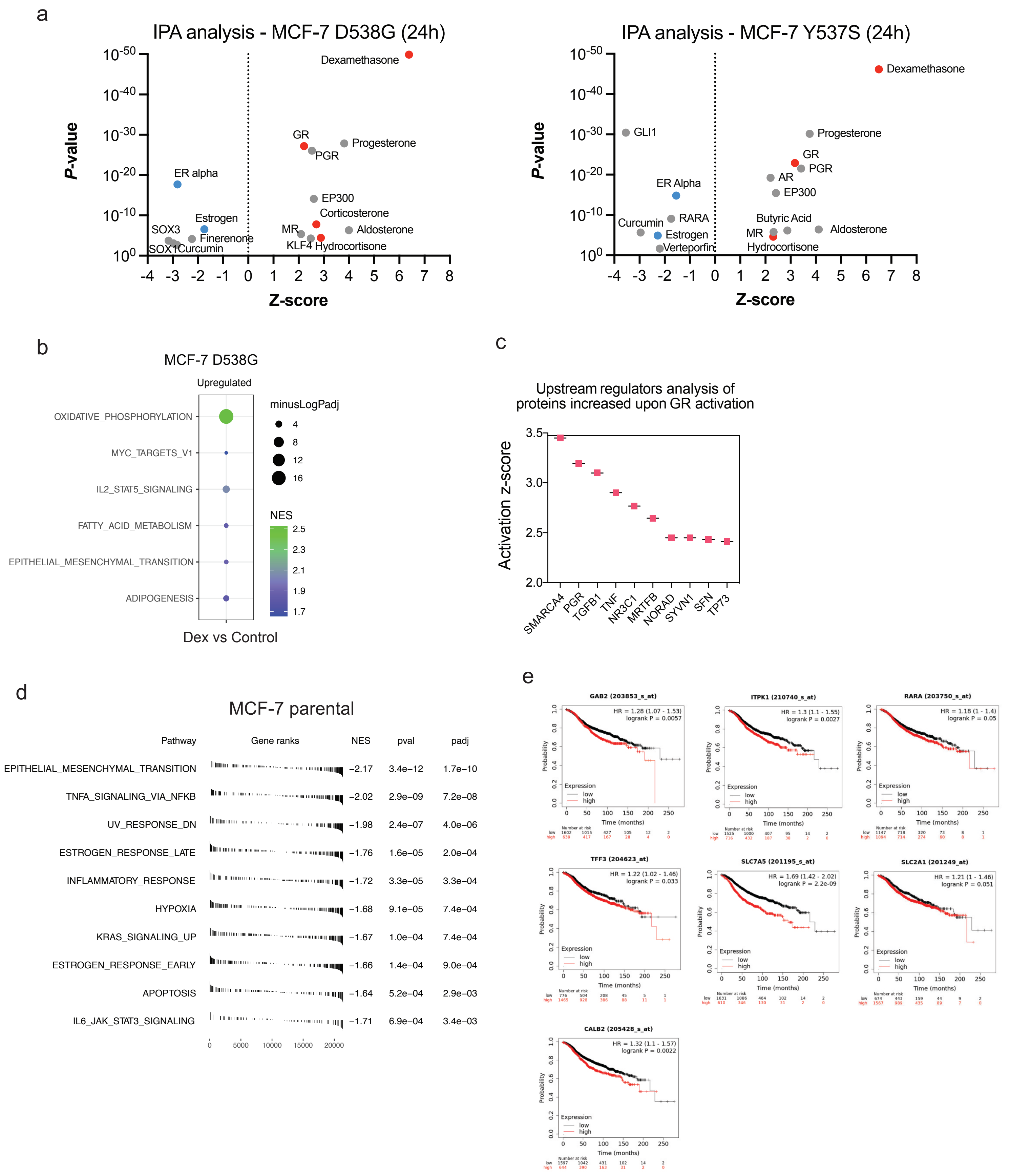
a
IPA analysis - MCF-7 D538G (24h)
P-value
Z-score
Dexamethasone
GR
Progesterone
PGR
ER alpha
EP300
Corticosterone
Estrogen
SOX3
Finerenone
SOX1
Curcumin
MR
Aldosterone
KLF4
Hydrocortisone
IPA analysis - MCF-7 Y537S (24h)
GLI1
Progesterone
GR
PGR
AR
ER Alpha
EP300
Curcumin
RARA
Butyric Acid
Estrogen
MR
Aldosterone
Verteporfin
Hydrocortisone
b
MCF-7 D538G
Upregulated
OXIDATIVE_PHOSPHORYLATION
MYC_TARGETS_V1
IL2_STAT5_SIGNALING
FATTY_ACID_METABOLISM
EPITHELIAL_MESENCHYMAL_TRANSITION
ADIPOGENESIS
minusLogPadj
NES
Dex vs Control
c
Upstream regulators analysis of proteins increased upon GR activation
Activation z-score
SMARCA4
PGR
TGFB1
TNF
NR3C1
MRTFB
NORAD
SYVN1
SFN
TP73
d
MCF-7 parental
Pathway
Gene ranks
NES
pval
padj
EPITHELIAL_MESENCHYMAL_TRANSITION −2.17 3.4e−12 1.7e−10
TNFA_SIGNALING_VIA_NFKB −2.02 2.9e−09 7.2e−08
UV_RESPONSE_DN −1.98 2.4e−07 4.0e−06
ESTROGEN_RESPONSE_LATE −1.76 1.6e−05 2.0e−04
INFLAMMATORY_RESPONSE −1.72 3.3e−05 3.3e−04
HYPOXIA −1.68 9.1e−05 7.4e−04
KRAS_SIGNALING_UP −1.67 1.0e−04 7.4e−04
ESTROGEN_RESPONSE_EARLY −1.66 1.4e−04 9.0e−04
APOPTOSIS −1.64 5.2e−04 2.9e−03
IL6_JAK_STAT3_SIGNALING −1.71 6.9e−04 3.4e−03
e
GAB2 (203853_s_at)
HR = 1.28 (1.07 - 1.53)
logrank P = 0.0057
ITPK1 (210740_s_at)
HR = 1.3 (1.1 - 1.55)
logrank P = 0.0027
RARA (203750_s_at)
HR = 1.18 (1 - 1.4)
logrank P = 0.05
TFF3 (204623_at)
HR = 1.22 (1.02 - 1.46)
logrank P = 0.033
SLC7A5 (201195_s_at)
HR = 1.69 (1.42 - 2.02)
logrank P = 2.2e-09
SLC2A1 (201249_at)
HR = 1.21 (1 - 1.46)
logrank P = 0.051
CALB2 (205428_s_at)
HR = 1.32 (1.1 - 1.57)
logrank P = 0.0022
Probability
Time (months)
Expression
low
high
Number at risk

Appendix Figure S3: Functional annotation of proteomics and RNA-Seq datasets.

a, Volcano plots showing the upstream regulators analysis of genes differentially regulated upon 24 h of GR activation in MCF-7 D538G and Y537S using IPA. **b**, Bubble plots depicting the normalized enrichment scores (NES) of upregulated (adjusted P-value < 0.05) Hallmark signature after GR activation in MCF-7 D538G models. **c,** Box plot showing the top 10 upstream regulators of proteins upregulated after GR activation as identified by ingenuity pathway analysis. **d,** Waterfall plot representing Gene Set Enrichment Analysis (GSEA) results of the top 10 downregulated Hallmark gene sets in MCF-7 parental cells after E2 and Dex treatment compared to E2 treatment alone (Microarray dataset; GSE79761). **e,** Kaplan Meier analyses of genes of the early E2-response gene signature and their clinical outcome (RFS) in ER+ HER2- breast cancer patients.

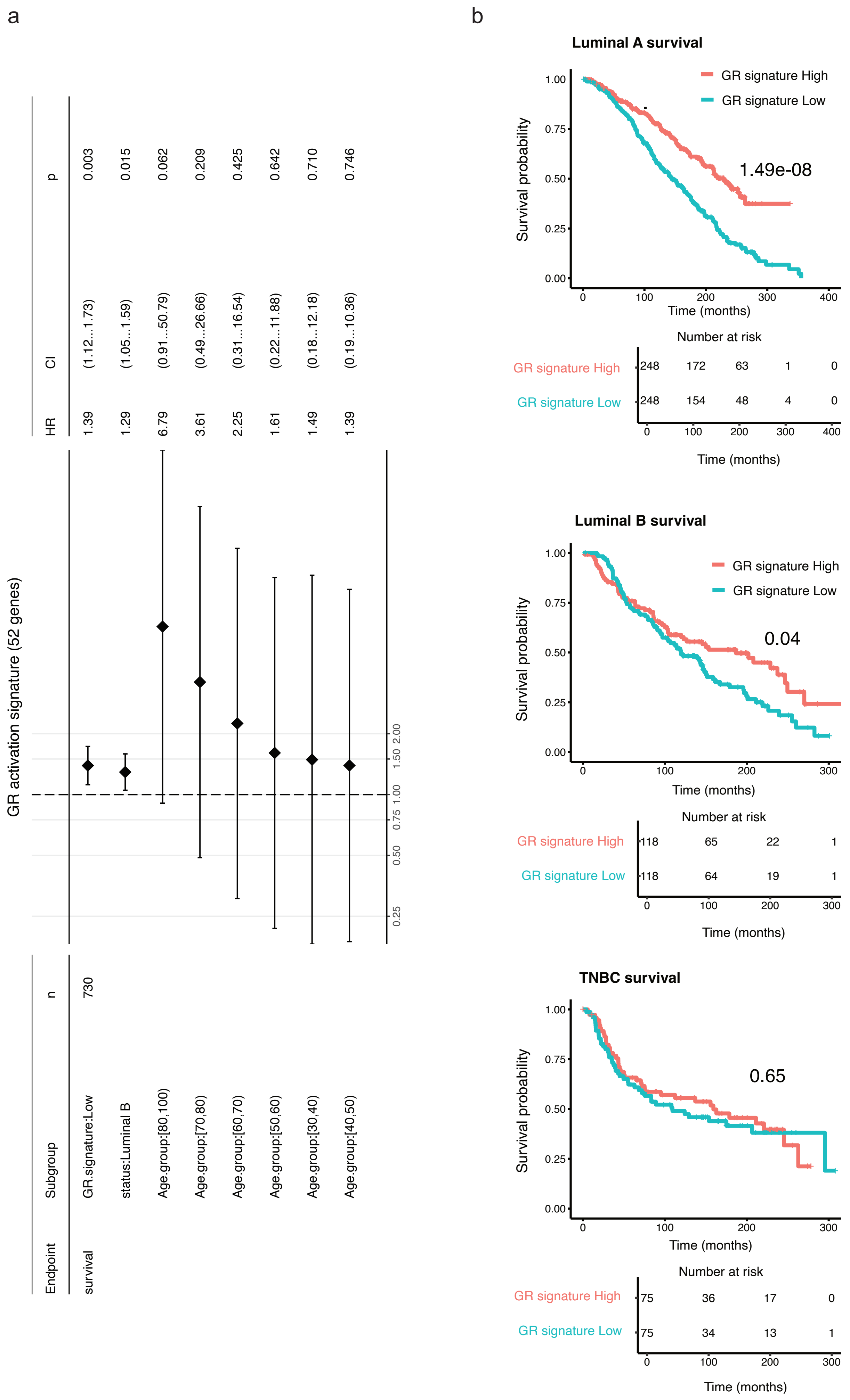
a
GR activation signature (52 genes)
Endpoint
Subgroup
n
HR
CI
p
survival
GR.signature:Low
730
1.39
(1.12...1.73)
0.003
status:Luminal B
1.29
(1.05...1.59)
0.015
Age.group:[80,100)
6.79
(0.91...50.79)
0.062
Age.group:[70,80)
3.61
(0.49...26.66)
0.209
Age.group:[60,70)
2.25
(0.31...16.54)
0.425
Age.group:[50,60)
1.61
(0.22...11.88)
0.642
Age.group:[30,40)
1.49
(0.18...12.18)
0.710
Age.group:[40,50)
1.39
(0.19...10.36)
0.746
0.25
0.50
0.75
1.00
1.50
2.00
b
Luminal A survival
GR signature High
GR signature Low
1.49e-08
Survival probability
Time (months)
Number at risk
GR signature High 248 172 63 1 0
GR signature Low 248 154 48 4 0
Luminal B survival
0.04
GR signature High 118 65 22 1
GR signature Low 118 64 19 1
TNBC survival
0.65
GR signature High 75 36 17 0
GR signature Low 75 34 13 1

Appendix Figure S4: Cox regression multivariate analyses using GR activity signature.

a, Multivariate survival analysis taking into account the expression of GR activity signature, the ER subtype (luminal A and B) and the age groups of the patients. n = number of patients, HR = hazard ration, CI = confidence interval, *p* = *p*-value. **b,** Kaplan Meier survival plots showing GR activity signature predictive value in Luminal A, Luminal B and TNBC breast cancer patients (METABRIC). Patients were stratified based on high and low GR activity signature score; Cox proportional hazard model with log-rank test.

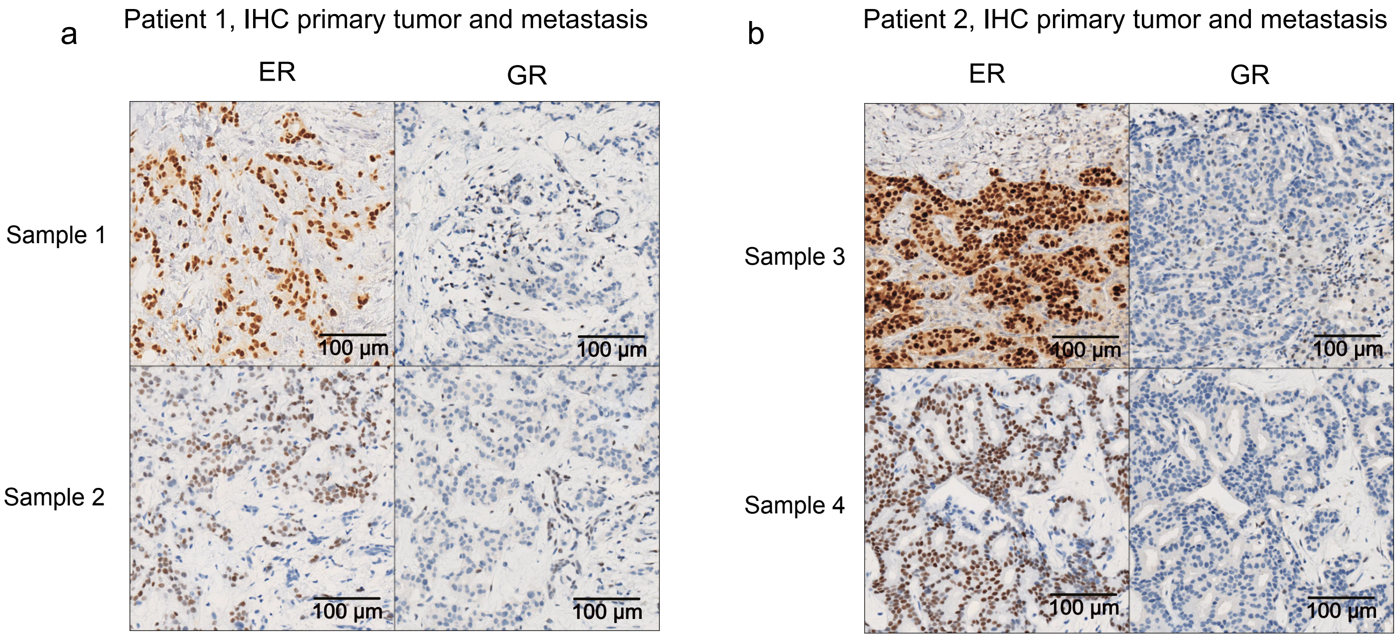

Appendix Figure S5: Immunohistochemistry of ER and GR proteins in ER+ patient samples.

a, b, Immunohistochemistry of representative primary tumor and liver metastases from ER+ metastatic patient samples stained for nuclear ER and GR; *n* = 43 samples. 20 x magnification, scale 100 M. The protein abundance of ER and GR was quantified and the H-Score calculated with the HALO software.
