# Supplementary figures and images for: Activated glucocorticoid receptor is an estrogen receptor silencer in ER+ metastatic breast cancer
Source: EMBO Mol Med. 2025 Nov 19;18(1):151–86. doi: 10.1038/s44321-025-00342-z (PMC12808765; doi:10.1038/s44321-025-00342-z)

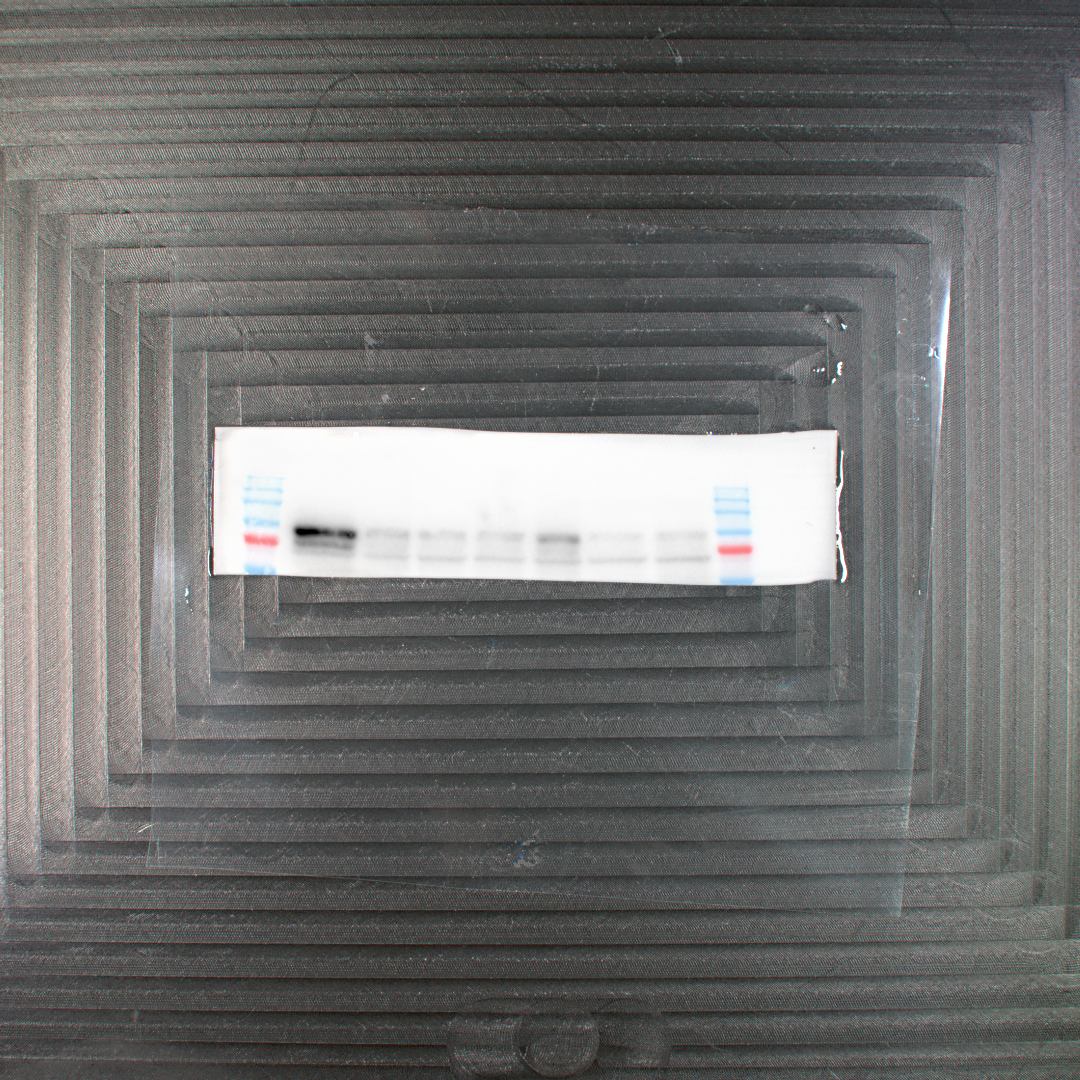

Supplement: Supplementary file 6 — Source data Fig. 1 [file 44321_2025_342_MOESM6_ESM.zip › Figure 1/1B/GR_super auto.Tif]

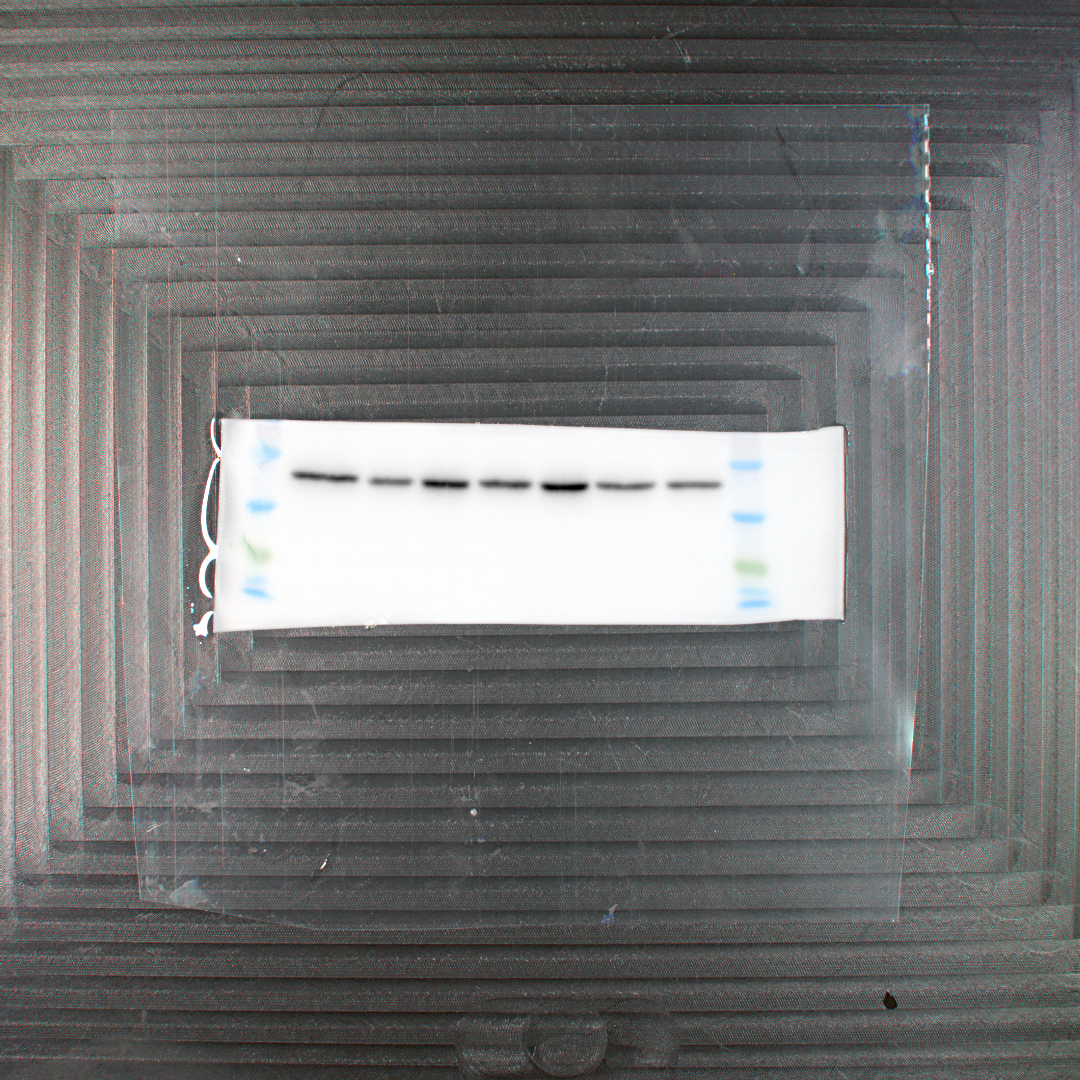

Supplement: Supplementary file 6 — Source data Fig. 1 [file 44321_2025_342_MOESM6_ESM.zip › Figure 1/1B/ERK_super auto.Tif]

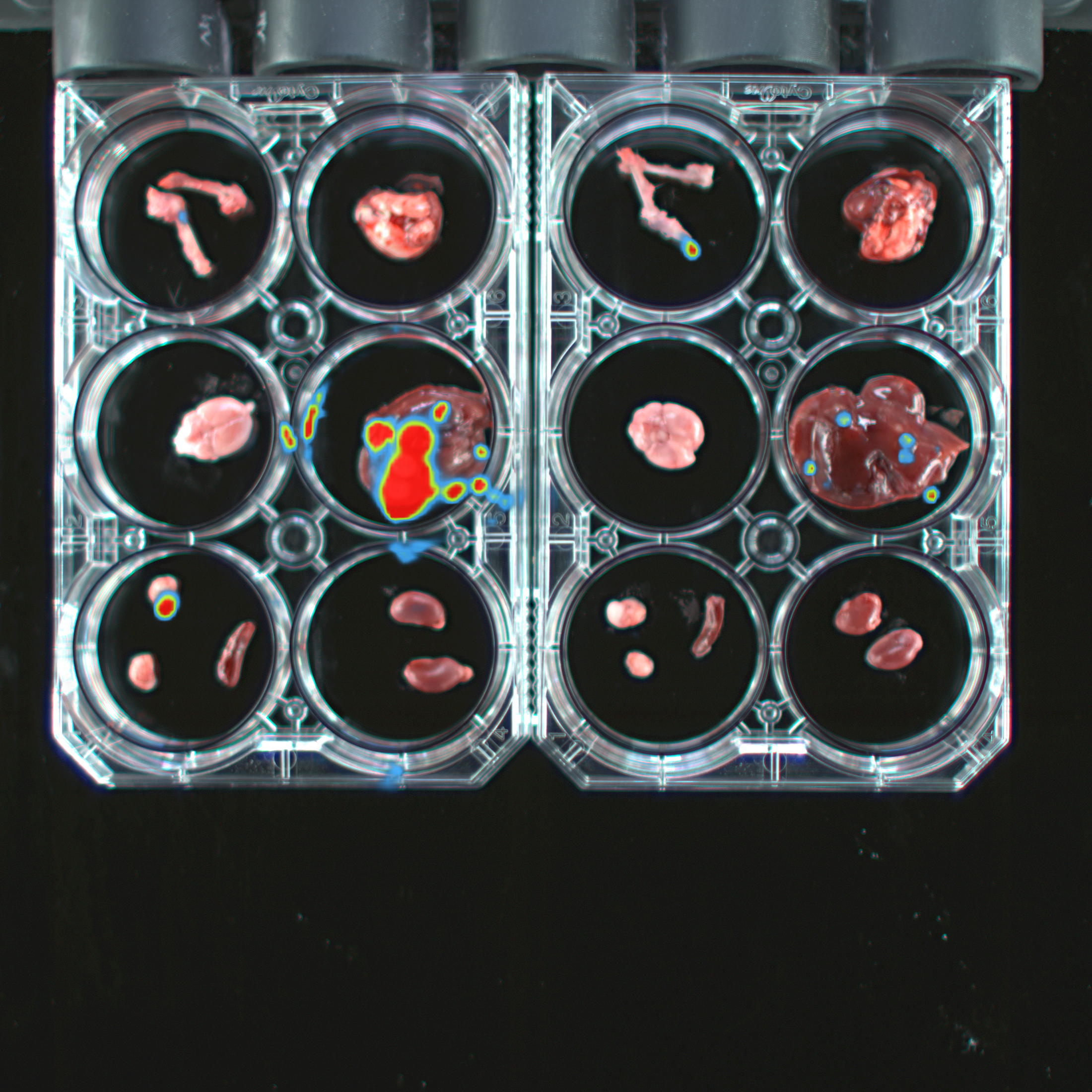

Supplement: Supplementary file 6 — Source data Fig. 1 [file 44321_2025_342_MOESM6_ESM.zip › Figure 1/1E/Control G2M2M3_30s.tif]

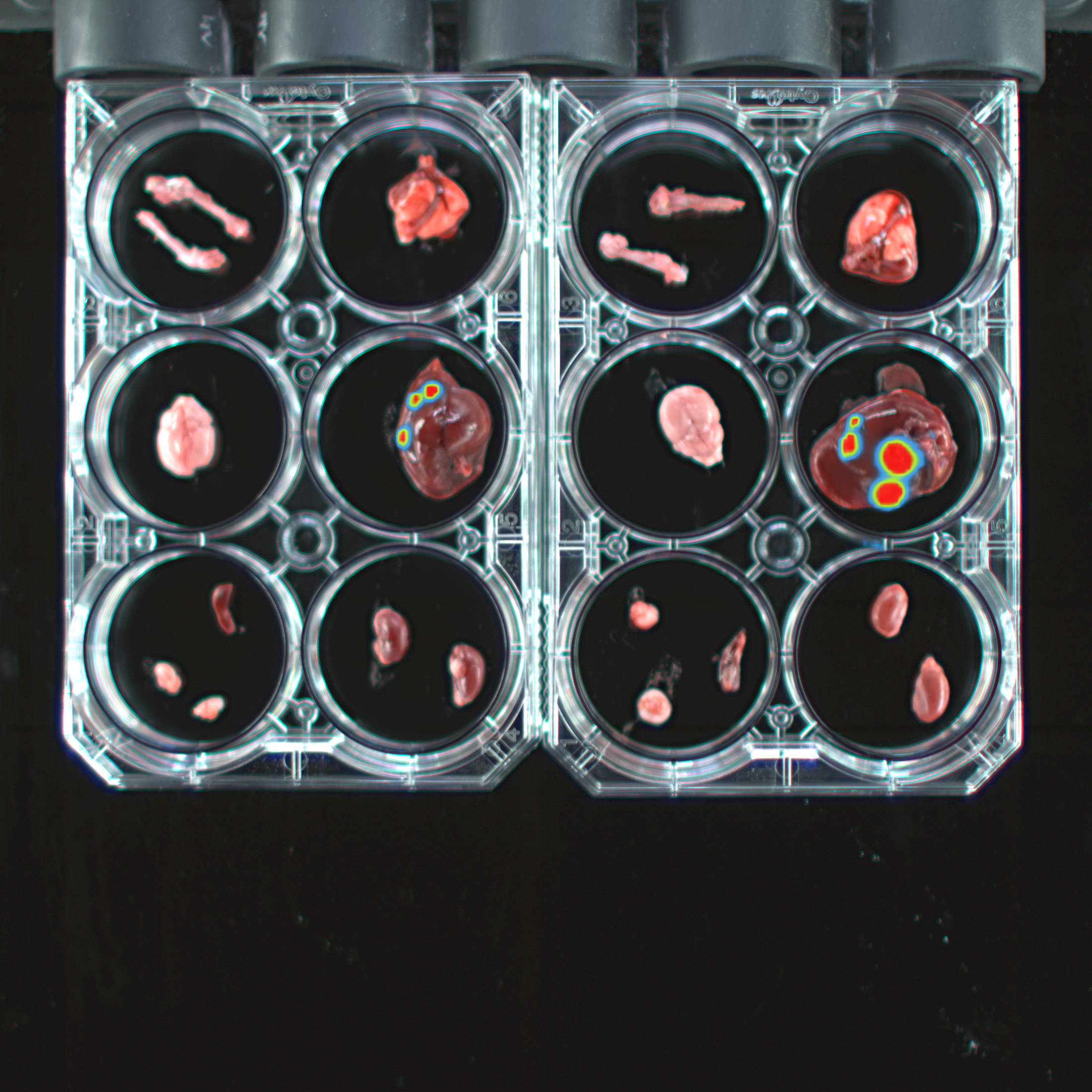

Supplement: Supplementary file 6 — Source data Fig. 1 [file 44321_2025_342_MOESM6_ESM.zip › Figure 1/1E/Dex G2M5_Control G2M1_30s.tif]

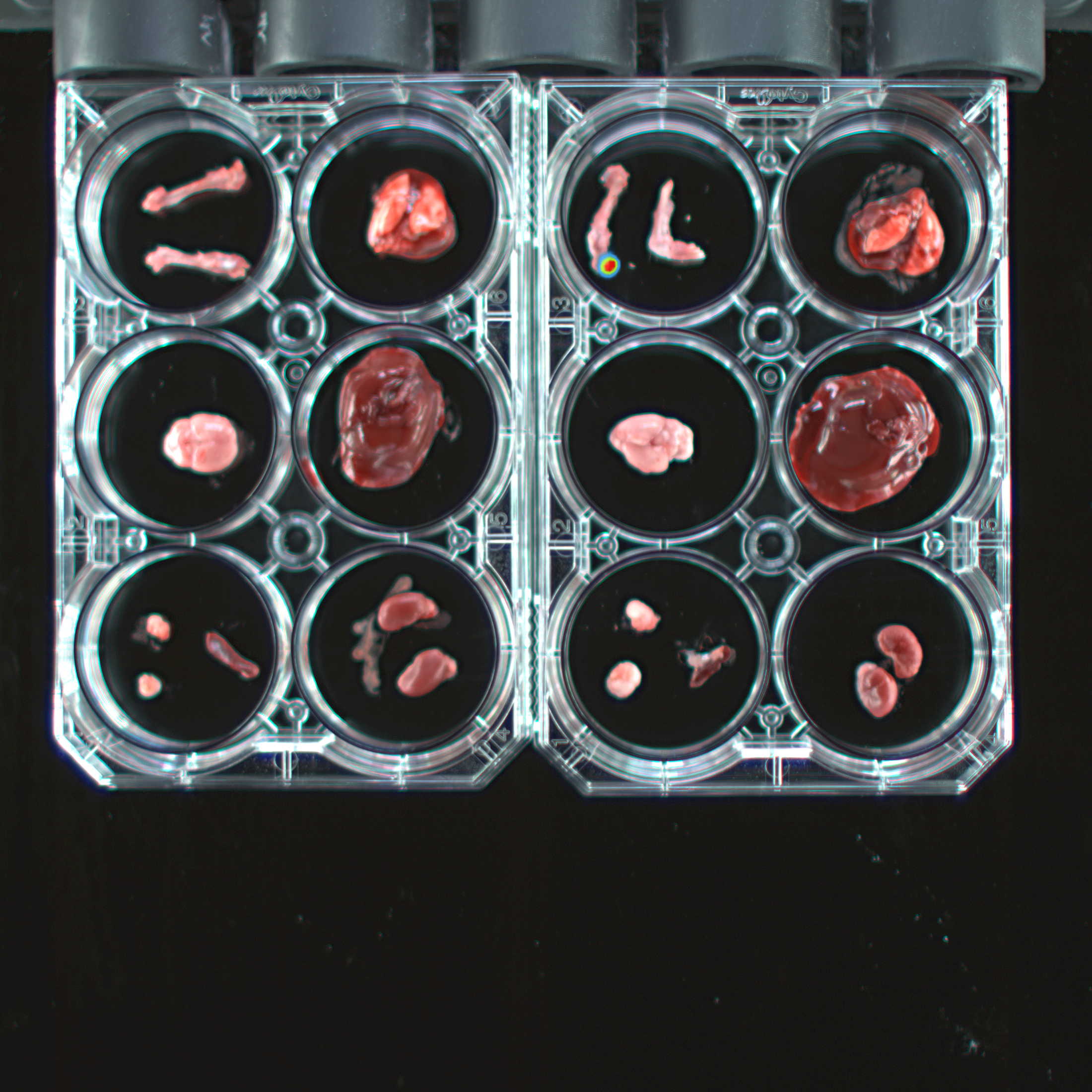

Supplement: Supplementary file 6 — Source data Fig. 1 [file 44321_2025_342_MOESM6_ESM.zip › Figure 1/1E/Dex G2M3M4_30s.tif]

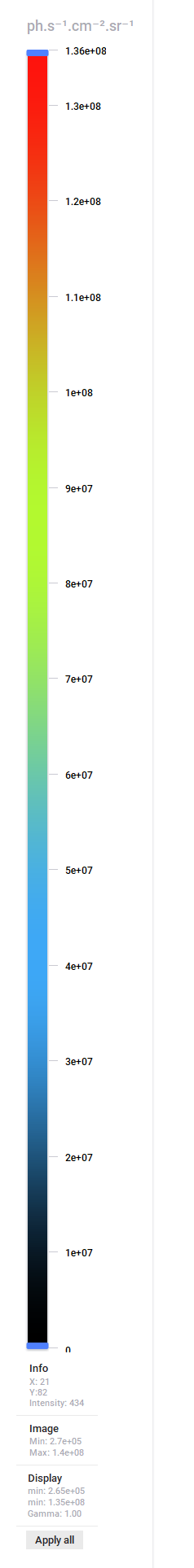

Supplement: Supplementary file 6 — Source data Fig. 1 [file 44321_2025_342_MOESM6_ESM.zip › Figure 1/1E/Scale for organs.PNG]

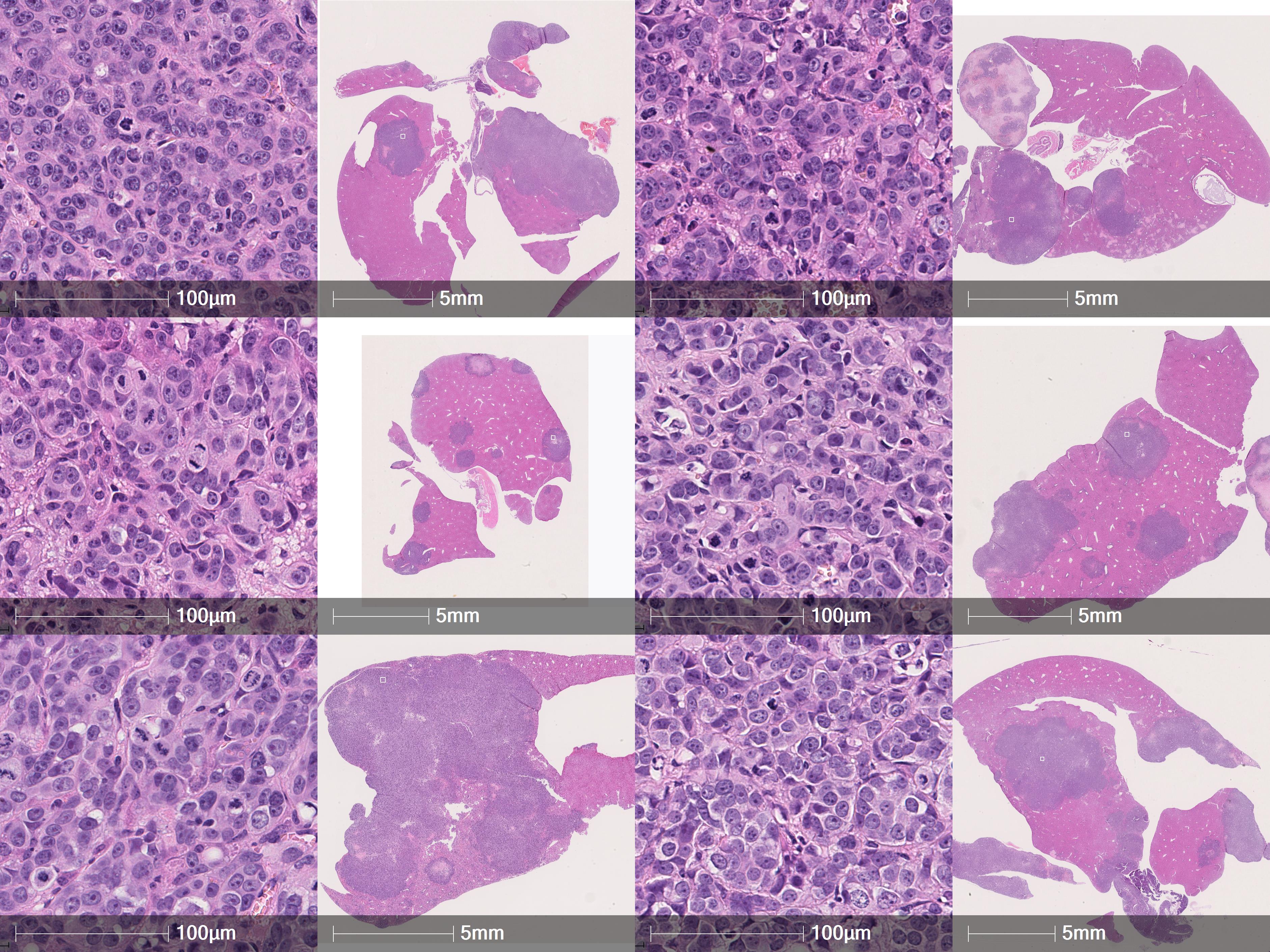

Supplement: Supplementary file 6 — Source data Fig. 1 [file 44321_2025_342_MOESM6_ESM.zip › Figure 1/1D/histology montage OMERO.jpg]

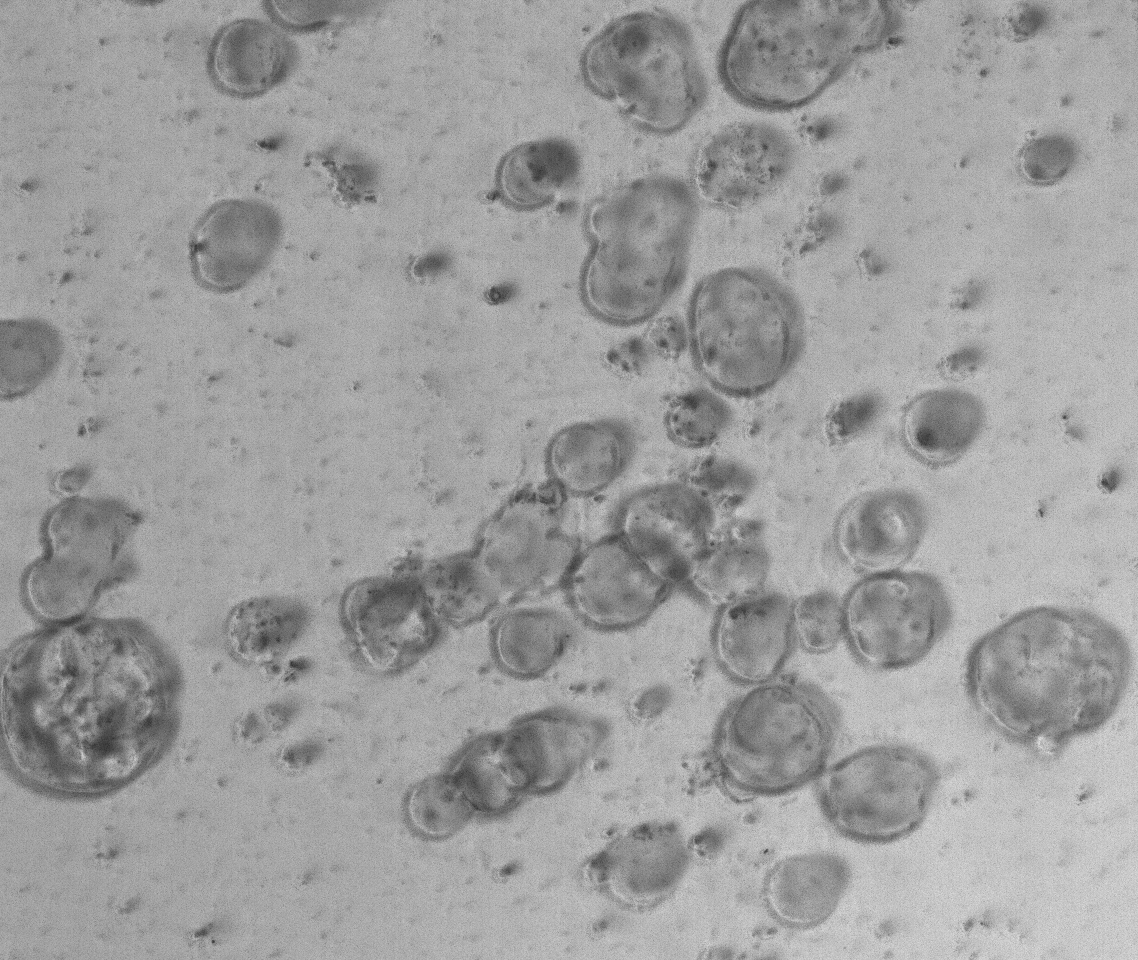

Supplement: Supplementary file 7 — Source data Fig. 2 [file 44321_2025_342_MOESM7_ESM.zip › Figure 2/2D-E/PDXO2 Dex_BF_I09.tif]

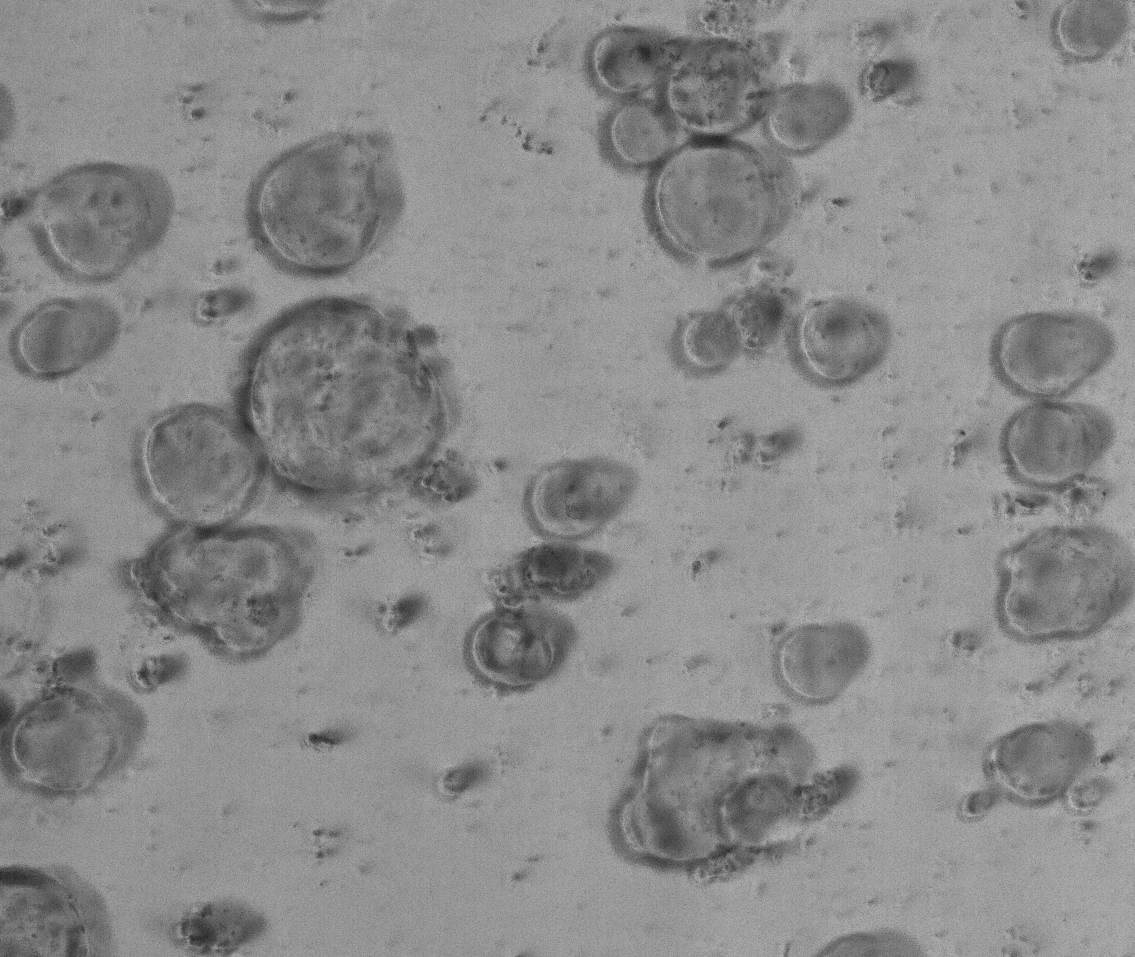

Supplement: Supplementary file 7 — Source data Fig. 2 [file 44321_2025_342_MOESM7_ESM.zip › Figure 2/2D-E/PDXO2 Ctrl BF_I08.tif]

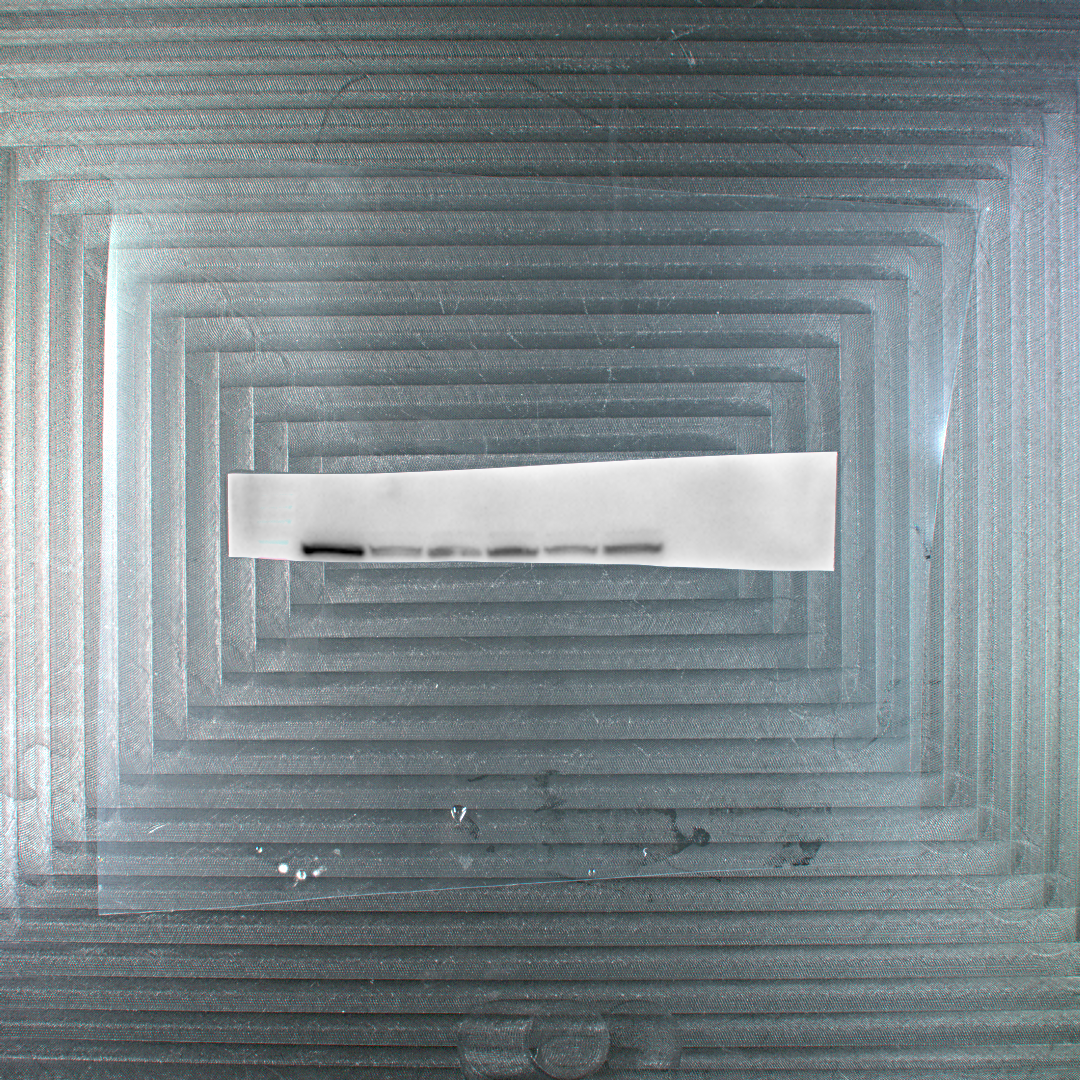

Supplement: Supplementary file 8 — Source data Fig. 3 [file 44321_2025_342_MOESM8_ESM.zip › Figure 3/3I/GR_super auto.Tif]

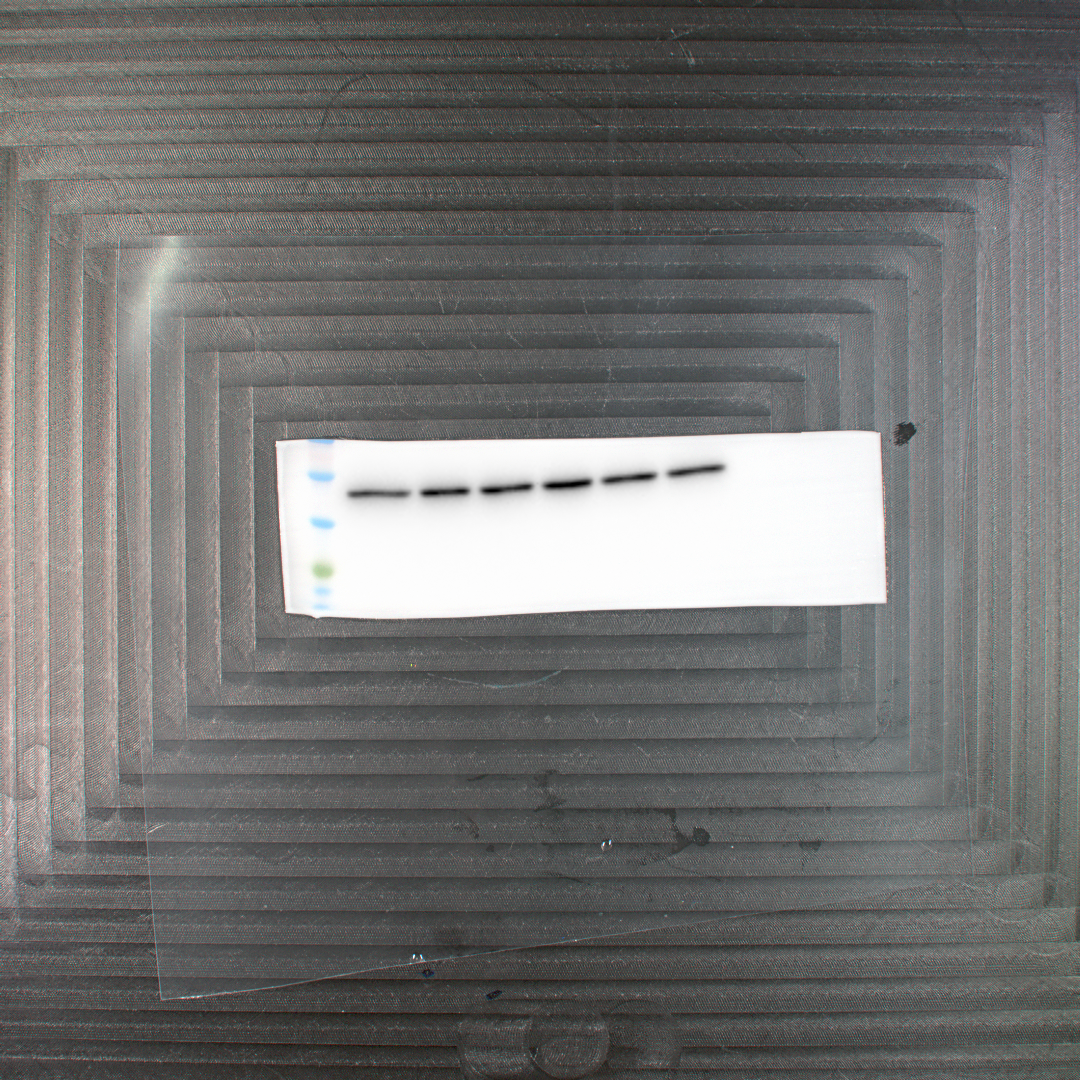

Supplement: Supplementary file 8 — Source data Fig. 3 [file 44321_2025_342_MOESM8_ESM.zip › Figure 3/3I/ERK_super auto.Tif]

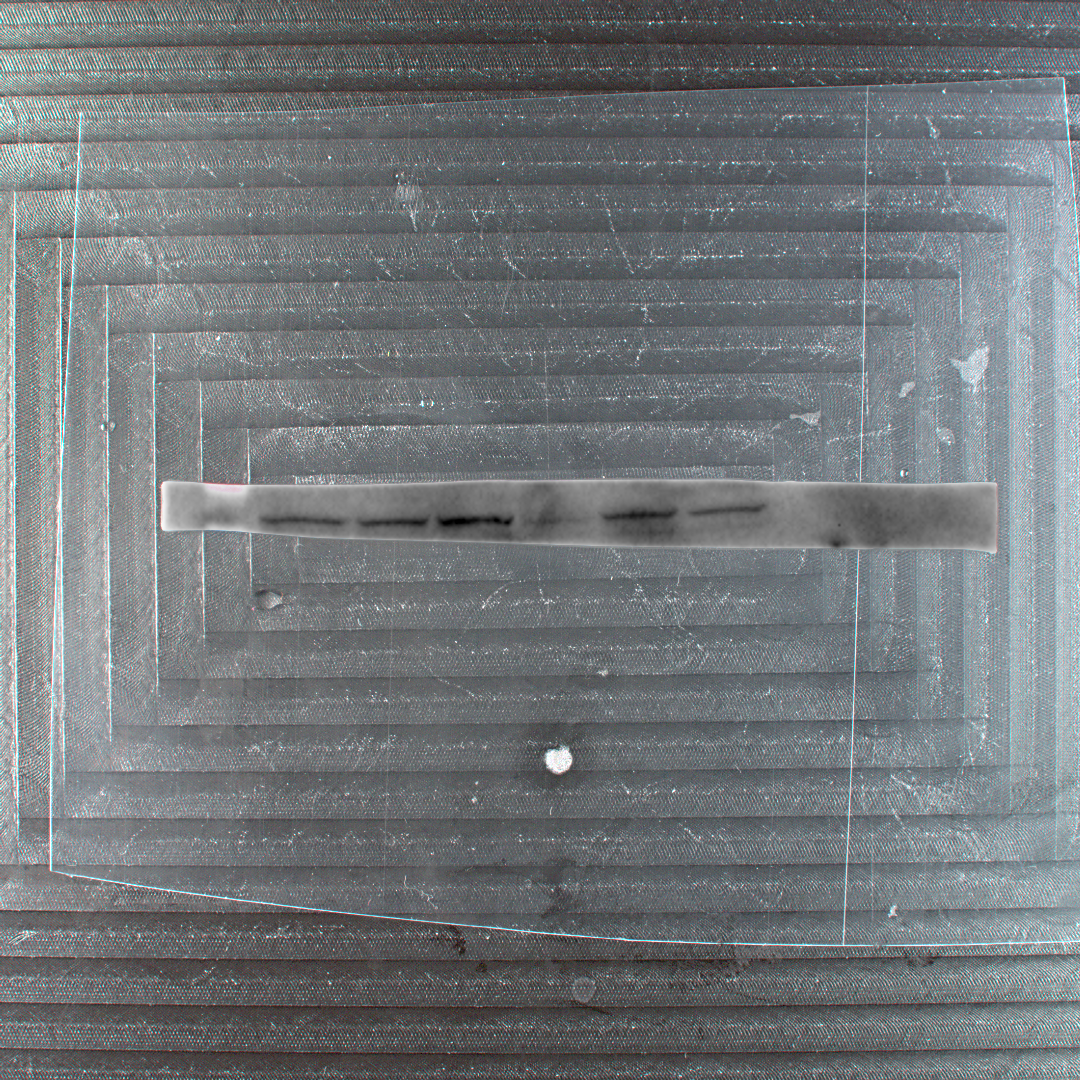

Supplement: Supplementary file 8 — Source data Fig. 3 [file 44321_2025_342_MOESM8_ESM.zip › Figure 3/3I/ER reblot 4_super auto.Tif]

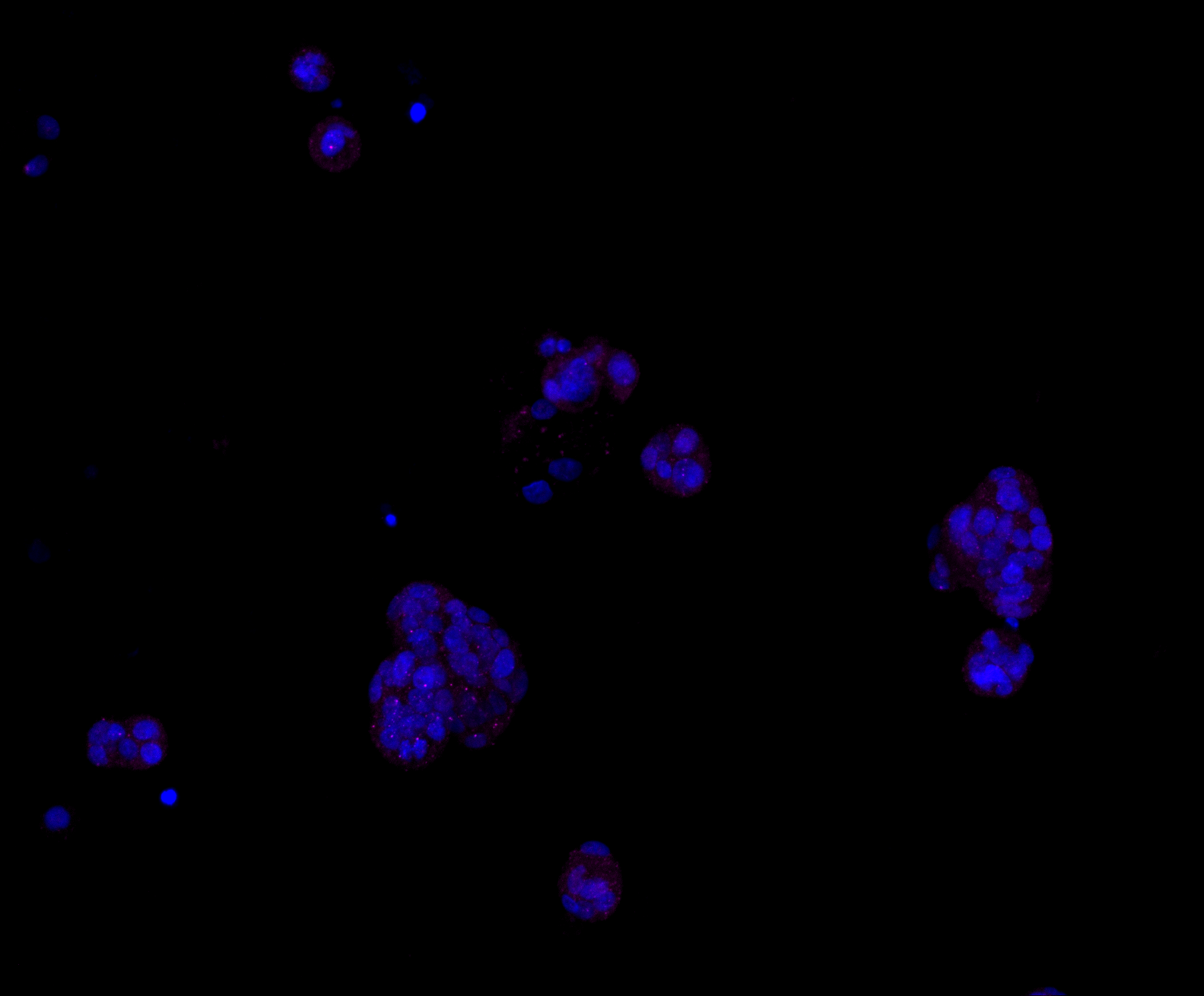

Supplement: Supplementary file 10 — Source data Fig. 5 [file 44321_2025_342_MOESM10_ESM.zip › Figure 5/5H/PDXO2/PDXO2_Dex_Fulves.tif]

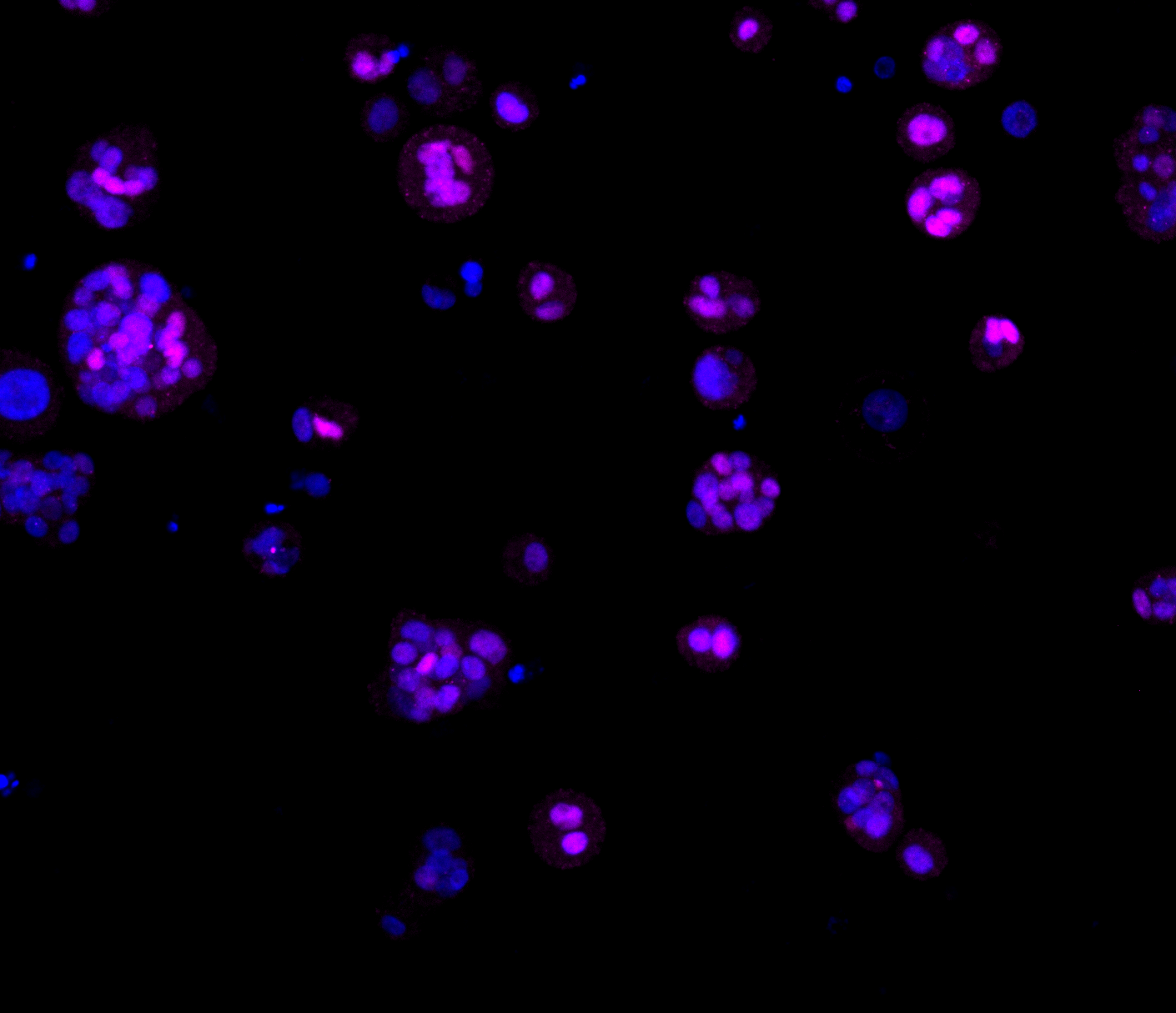

Supplement: Supplementary file 10 — Source data Fig. 5 [file 44321_2025_342_MOESM10_ESM.zip › Figure 5/5H/PDXO2/PDXO2_control.tif]

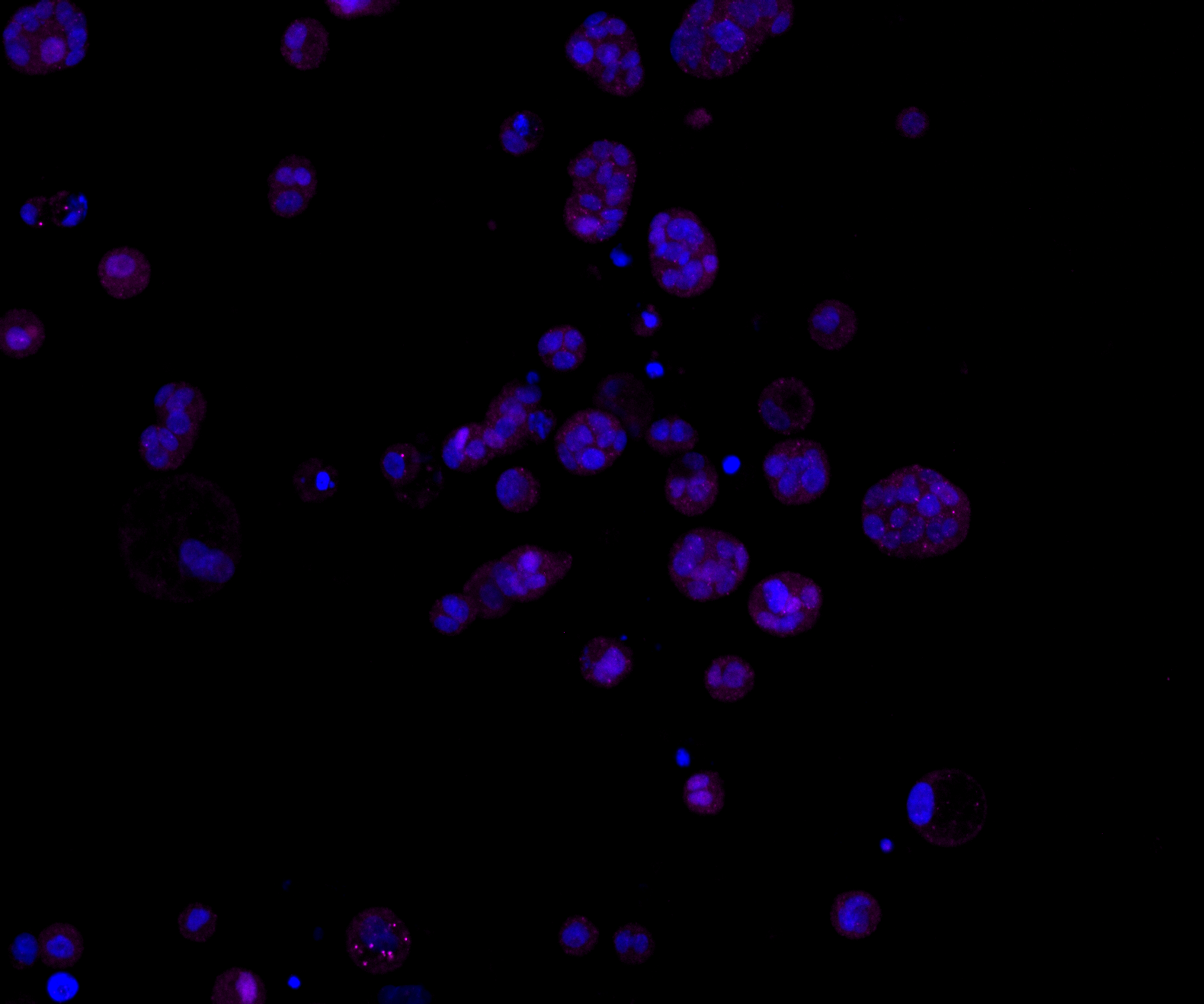

Supplement: Supplementary file 10 — Source data Fig. 5 [file 44321_2025_342_MOESM10_ESM.zip › Figure 5/5H/PDXO2/PDXO2_Dex.tif]

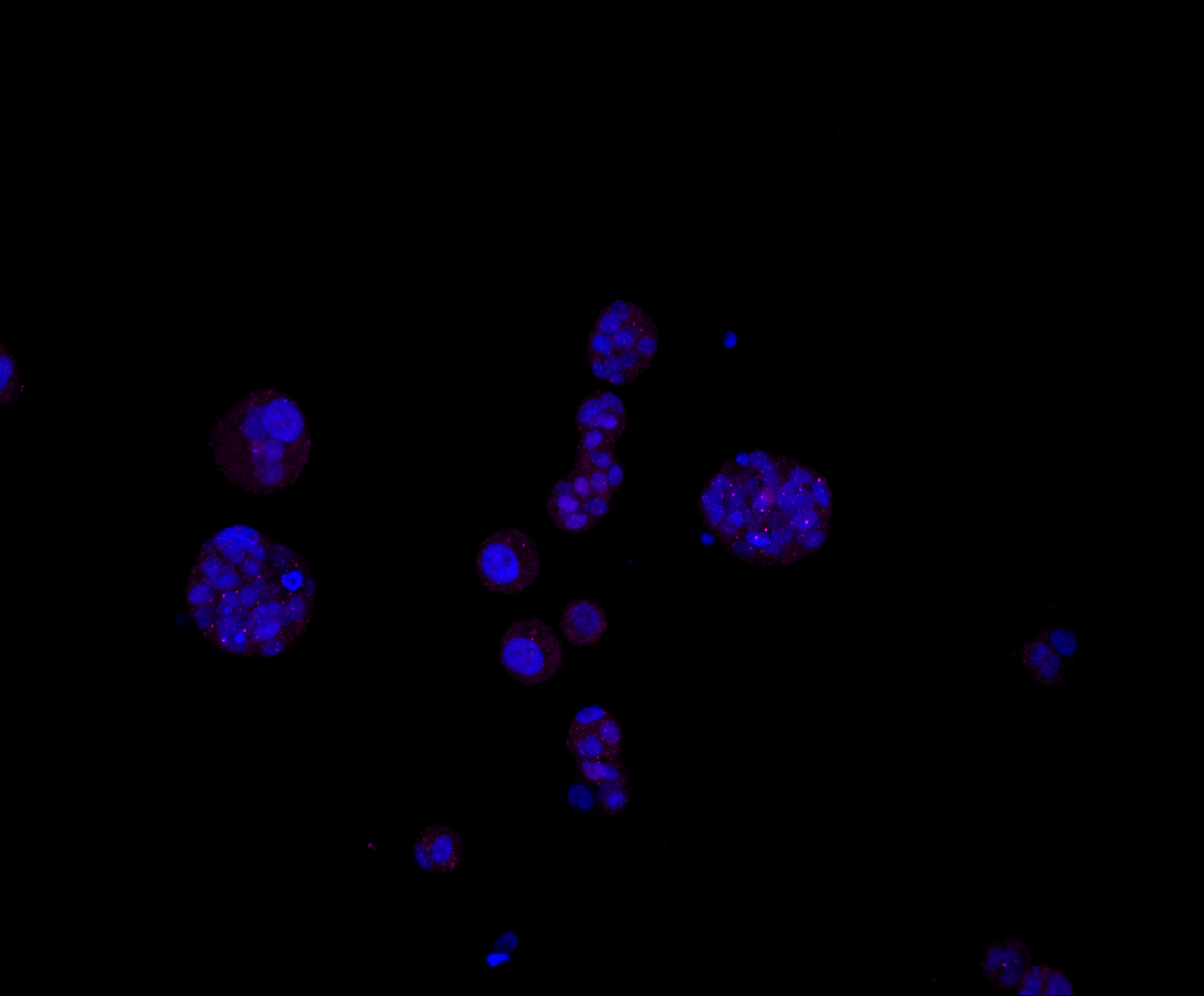

Supplement: Supplementary file 10 — Source data Fig. 5 [file 44321_2025_342_MOESM10_ESM.zip › Figure 5/5H/PDXO2/PDXO2_Fulves.tif]

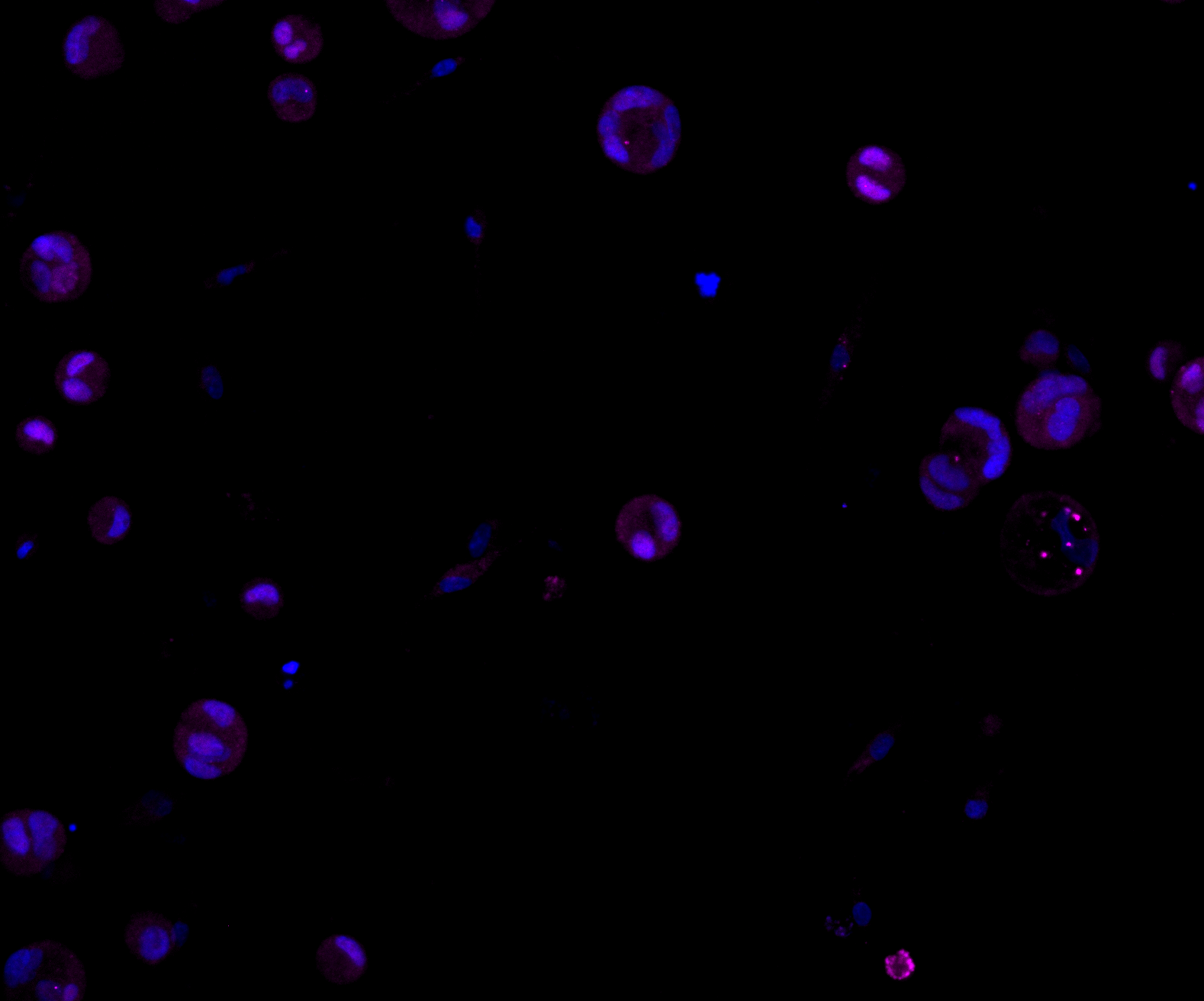

Supplement: Supplementary file 10 — Source data Fig. 5 [file 44321_2025_342_MOESM10_ESM.zip › Figure 5/5H/PDO1/PDO1_Dex_Fulves.tif]

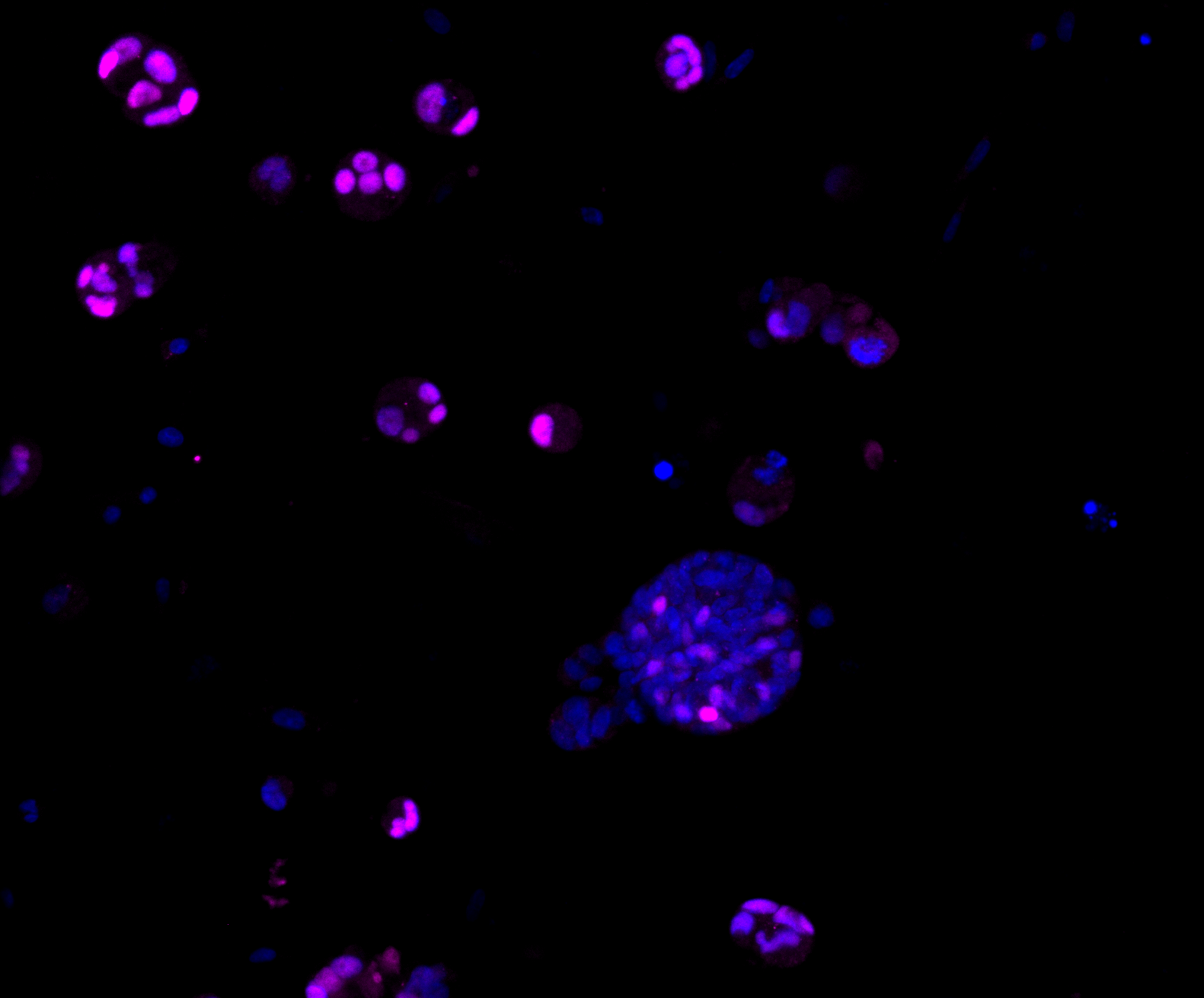

Supplement: Supplementary file 10 — Source data Fig. 5 [file 44321_2025_342_MOESM10_ESM.zip › Figure 5/5H/PDO1/PDO1_Dex.tif]

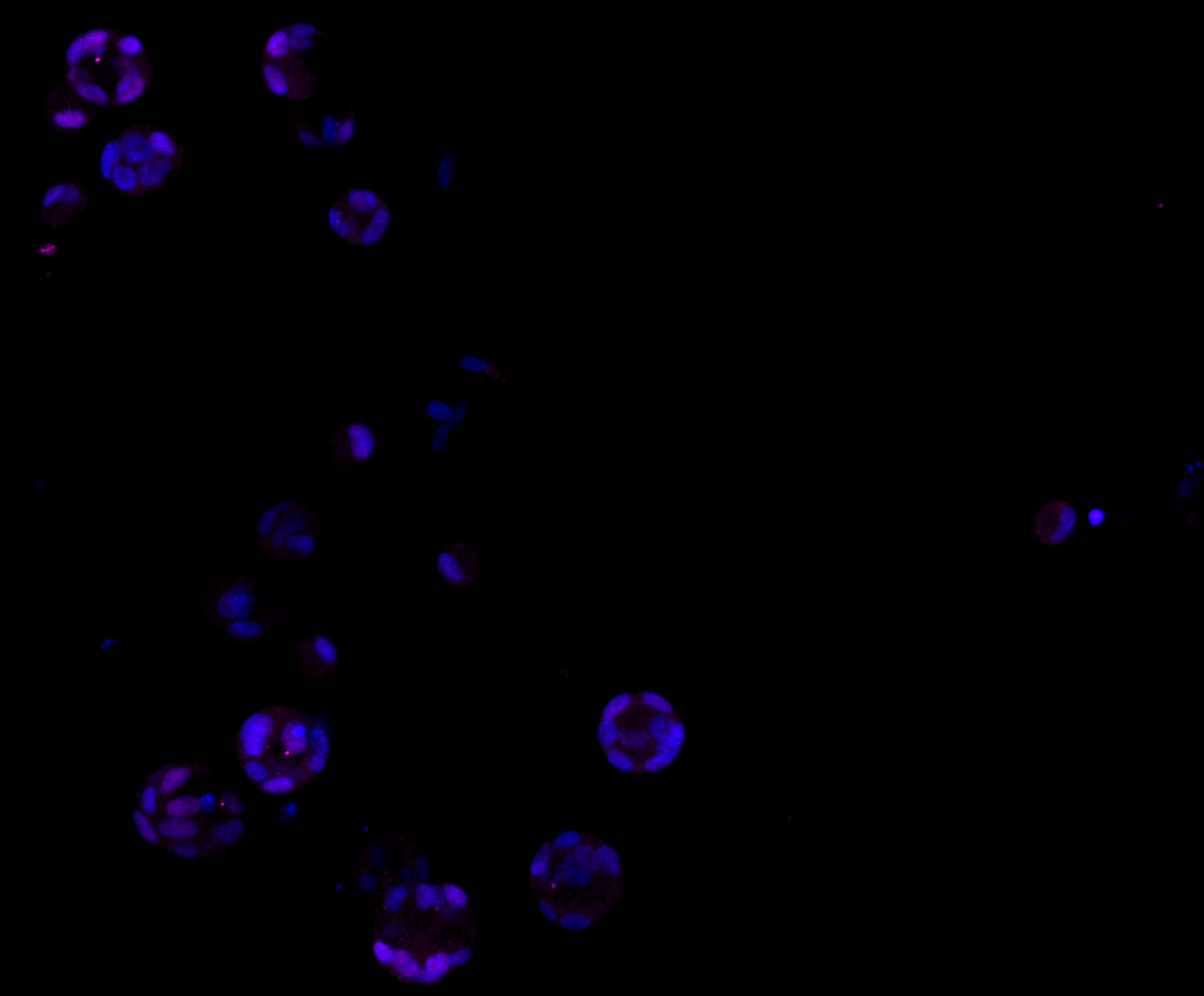

Supplement: Supplementary file 10 — Source data Fig. 5 [file 44321_2025_342_MOESM10_ESM.zip › Figure 5/5H/PDO1/PDO1_Fulves.tif]

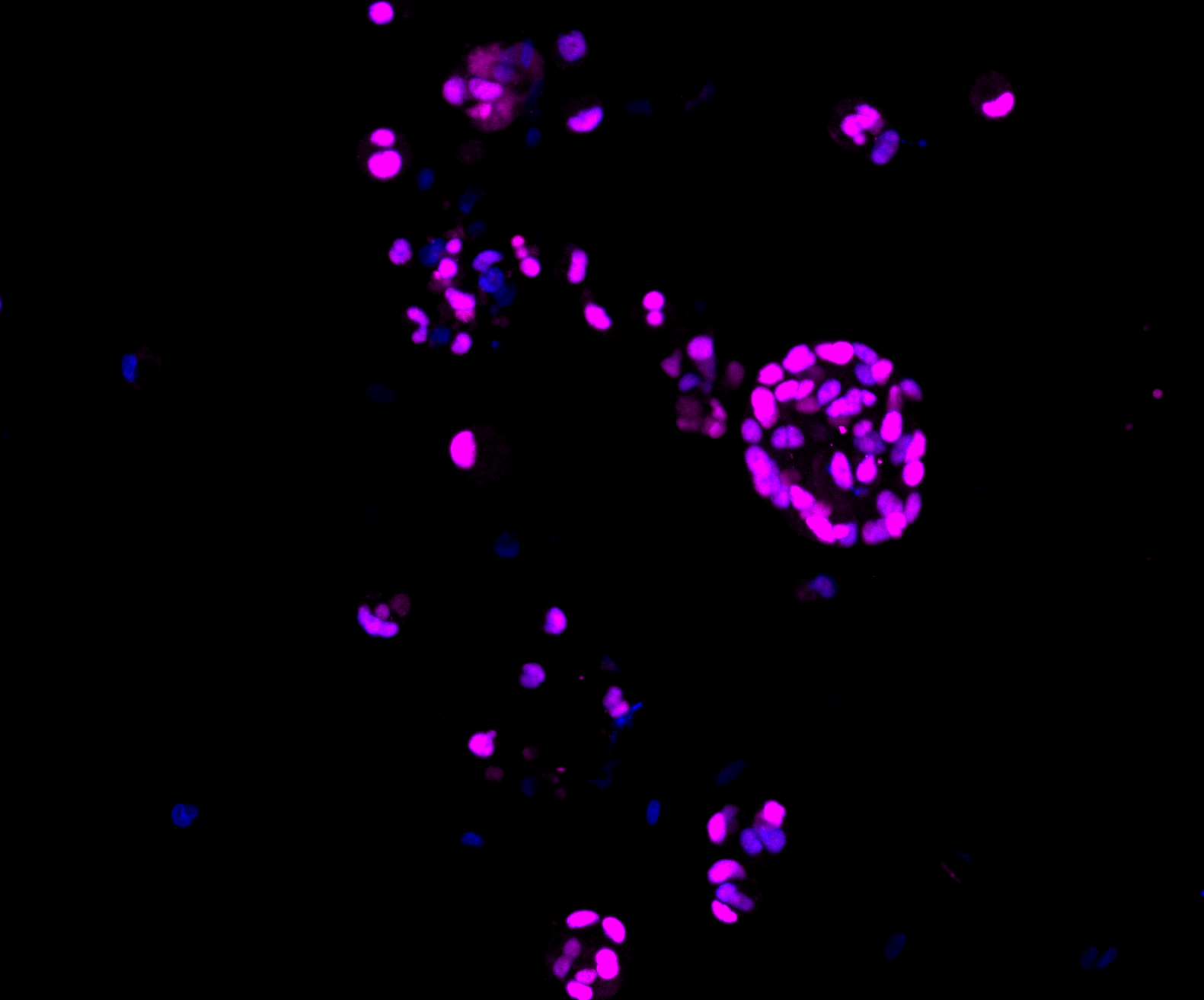

Supplement: Supplementary file 10 — Source data Fig. 5 [file 44321_2025_342_MOESM10_ESM.zip › Figure 5/5H/PDO1/PDO1_control.tif]
