# Supplementary material for: Activated glucocorticoid receptor is an estrogen receptor silencer in ER+ metastatic breast cancer
Source: EMBO Mol Med. 2025 Nov 19;18(1):151–86. doi: 10.1038/s44321-025-00342-z (PMC12808765; doi:10.1038/s44321-025-00342-z)
Supplement: Supplementary file 11 — Expanded View Figures [file 44321_2025_342_MOESM11_ESM.pdf]

Expanded View Figures

Figure EV1. Nuclear translocation and genetic knockdown of GR in *ESR1* mutant models.

(**A**) Immunoblots showing GR and ER abundance in *ESR1* wild-type *versus* mutant ER+ cells. ERK2 was used as a loading control. (**B**) Representative immunofluorescence images of MCF-7 D538G cells showing GR nuclear translocation after activation by Dex for 1 h. Scale 200 μM. (**C**) Representative immunohistochemistry images of metastatic livers from MCF-7 D538G xenografts showing the GR nuclear translocation after activation by Dex for 16 h prior to sacrifice; ×40 magnification, scale 50 μm. (**D**) Bar graph representing mRNA levels of *SGK1* in MCF-7 D538G cells after GR activation for 72 h; $n = 3$ biological replicates. Two-tailed *t* test. Data are presented as mean ± SD. (**E**) Bar graph depicting mRNA levels of *NR3C1* in cells transduced with control shRNA or shRNAs targeting GR (shGR1 and shGR2). $n = 3$ to 4 biological replicates. Two-tailed *t* test. (**F**) Bar graph depicting mRNA levels of *SGK1* after Dex treatment in cells transduced with control shRNA or shRNAs targeting GR (shGR1 and shGR2); $n = 3$ biological replicates. Two-tailed *t* test. (**G**) Bioluminescence imaging of mice intravenously injected with MCF-7 D538G cells transduced with control shRNA or shRNAs targeting GR (shGR1 and shGR2); $n = 5$ mice per group. Imaging was performed with Newton Vilber. (**H**) Graph representing estimations of metastatic burden in the whole body of mice over time. (**I**) Representative bioluminescence images of MCF-7 D538G liver metastases harvested at day 47. $n =$ 9–10 mice per group. Imaging of livers was performed with Newton Vilber.

© The Author(s)

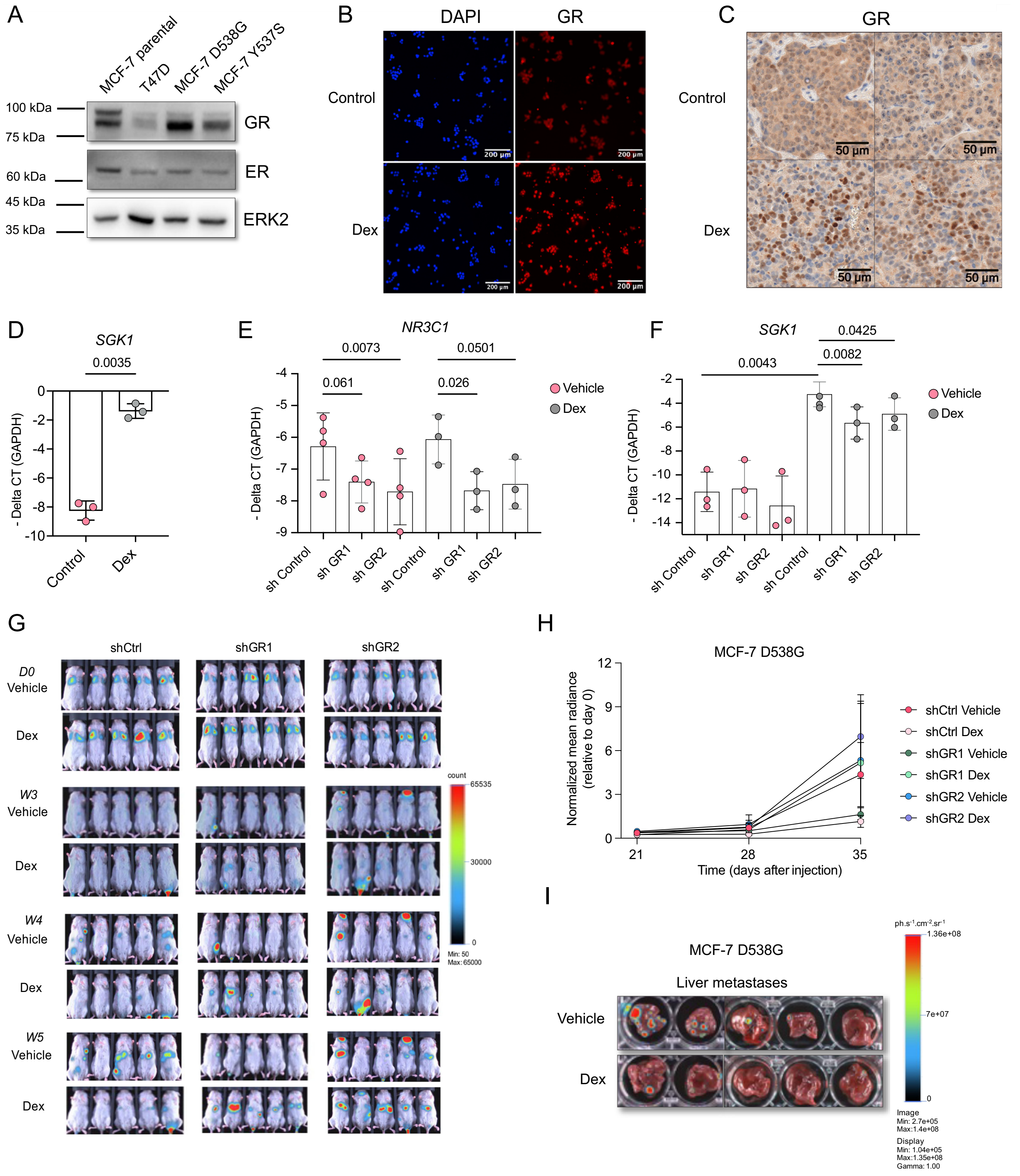
A
MCF-7 parental
T47D
MCF-7 D538G
MCF-7 Y537S
100 kDa
75 kDa
GR
60 kDa
ER
45 kDa
35 kDa
ERK2
B
DAPI
GR
Control
Dex
C
GR
Control
Dex
50 µm
D
SGK1
0.0035
- Delta CT (GAPDH)
Control
Dex
E
NR3C1
0.0073
0.0501
0.061
0.026
Vehicle
Dex
sh Control
sh GR1
sh GR2
F
SGK1
0.0425
0.0082
0.0043
G
shCtrl
shGR1
shGR2
D0
Vehicle
Dex
W3
W4
W5
count
65535
30000
Min: 50
Max: 65000
H
MCF-7 D538G
Normalized mean radiance (relative to day 0)
Time (days after injection)
shCtrl Vehicle
shCtrl Dex
shGR1 Vehicle
shGR1 Dex
shGR2 Vehicle
shGR2 Dex
I
MCF-7 D538G
Liver metastases
Vehicle
Dex
1.36e+08
7e+07
Image
Min: 2.7e+05
Max:1.4e+08
Display
Min: 1.04e+05
Max:1.35e+08
Gamma: 1.00

© The Author(s)

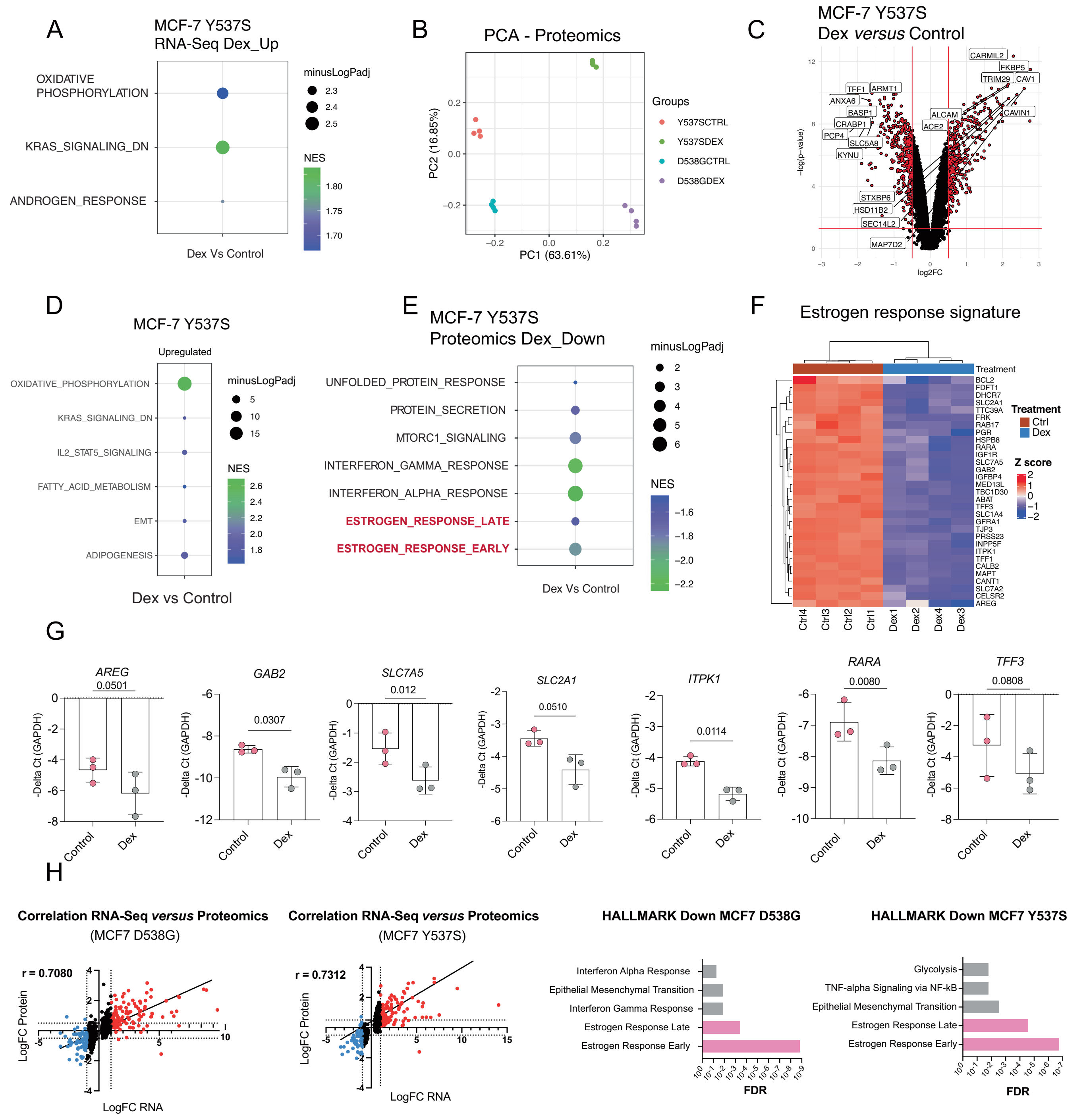
A
MCF-7 Y537S
RNA-Seq Dex_Up
OXIDATIVE PHOSPHORYLATION
KRAS_SIGNALING_DN
ANDROGEN_RESPONSE
minusLogPadj
NES
Dex Vs Control
B
PCA - Proteomics
PC1 (63.61%)
PC2 (16.85%)
Groups
Y537SCTRL
Y537SDEX
D538GCTRL
D538GDEX
C
MCF-7 Y537S
Dex versus Control
log2FC
−log(p−value)
D
MCF-7 Y537S
Upregulated
OXIDATIVE_PHOSPHORYLATION
KRAS_SIGNALING_DN
IL2_STAT5_SIGNALING
FATTY_ACID_METABOLISM
EMT
ADIPOGENESIS
Dex vs Control
E
MCF-7 Y537S
Proteomics Dex_Down
UNFOLDED_PROTEIN_RESPONSE
PROTEIN_SECRETION
MTORC1_SIGNALING
INTERFERON_GAMMA_RESPONSE
INTERFERON_ALPHA_RESPONSE
ESTROGEN_RESPONSE_LATE
ESTROGEN_RESPONSE_EARLY
Dex Vs Control
F
Estrogen response signature
Treatment
Ctrl
Dex
Z score
G
AREG GAB2 SLC7A5 SLC2A1 ITPK1 RARA TFF3
-Delta Ct (GAPDH)
Control
Dex
H
Correlation RNA-Seq versus Proteomics (MCF7 D538G)
r = 0.7080
Correlation RNA-Seq versus Proteomics (MCF7 Y537S)
r = 0.7312
LogFC Protein
LogFC RNA
HALLMARK Down MCF7 D538G
Interferon Alpha Response
Epithelial Mesenchymal Transition
Interferon Gamma Response
Estrogen Response Late
Estrogen Response Early
HALLMARK Down MCF7 Y537S
Glycolysis
TNF-alpha Signaling via NF-kB
Epithelial Mesenchymal Transition
Estrogen Response Late
Estrogen Response Early
FDR

© The Author(s)

Figure EV2. Transcriptomic and proteomic profiling reveal downregulation of E2 response signatures upon Dex.

(**A**) Bubble plot depicting the Hallmark signatures enriched in genes upregulated upon 24 h Dex treatment in MCF-7 Y537S model. Normalized enrichment score (NES) and – Log adjusted-value are indicated. (**B**) Principal Component Analysis showing treatment group-based separation of MCF-7 D538G and Y537S cells treated with Dex or vehicle for 7 days and analyzed by proteomics; $n = 4$ technical replicates per condition. (**C**) Volcano plot of differentially abundant proteins (adjusted P value < 0.05 and |logFC | > 0.5) in Dex- versus vehicle-treated MCF-7 Y537S cells. Empirical Bayes moderated t-statistics with Benjamini–Hochberg correction. (**D**) Bubble plot depicting the NES of upregulated (adjusted P value < 0.05) Hallmark signature after GR activation in MCF-7 Y537S model. Kolmogovo–Smirnov-like running-sum statistics with permutation test and Benjamini–Hochberg correction. (**E**) Bubble plot depicting the NES of downregulated (adjusted P value < 0.05) Hallmark signature after GR activation in MCF-7 Y537S model. Kolmogorov–Smirnov-like running-sum statistics with permutation test and Benjamini–Hochberg correction. (**F**) Heatmap depicting the differentially abundant early estrogen response proteins (Hallmark gene set) downregulated after GR activation in MCF-7 D538G model; $n = 4$ technical replicates per condition. (**G**) Scatter dot plots showing mRNA levels of the early E2-response gene signature in MCF-7 D538G cells treated for 72 h with Dex or vehicle; $n = 3$ biological replicates. Two-tailed t test. Data are presented as mean ± SD. (**H**) Left panels: correlation between genes and proteins found differentially regulated in the RNA-Seq and proteomics datasets for both MCF-7 D538G and Y537S models. "r" indicates Pearson coefficient, and the line represents simple linear regression. Right panels: Hallmark functional annotation of the genes and proteins whose expression was downregulated upon Dex in both RNA-Seq and proteomics datasets, for both models.

 © The Author(s)

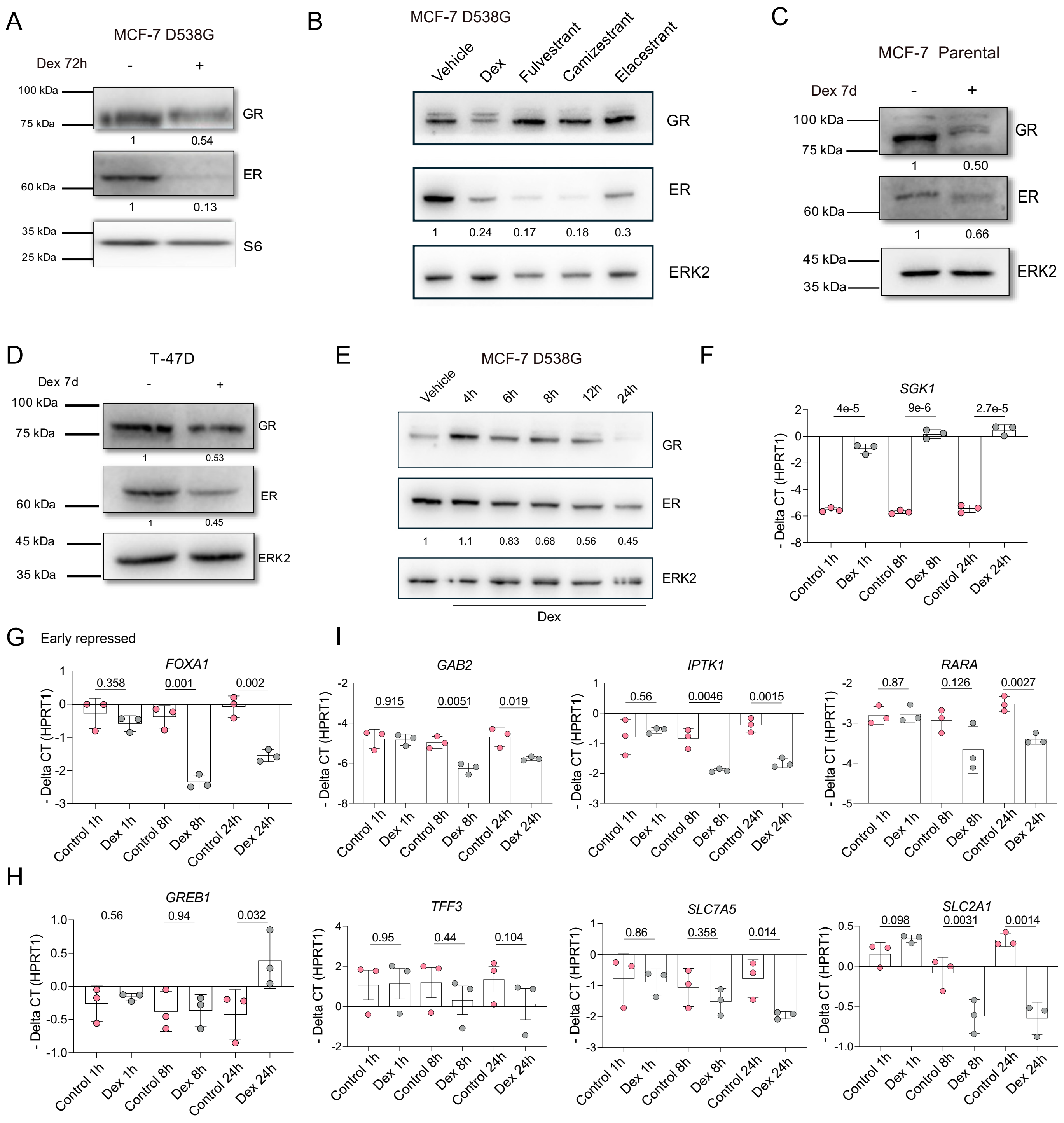
A
MCF-7 D538G
Dex 72h - +
GR
1 0.54
ER
1 0.13
S6
B
MCF-7 D538G
Vehicle Dex Fulvestrant Camizestrant Elacestrant
GR
ER
1 0.24 0.17 0.18 0.3
ERK2
C
MCF-7 Parental
Dex 7d - +
GR
1 0.50
ER
1 0.66
ERK2
D
T-47D
Dex 7d - +
GR
1 0.53
ER
1 0.45
ERK2
E
MCF-7 D538G
Vehicle 4h 6h 8h 12h 24h
GR
ER
1 1.1 0.83 0.68 0.56 0.45
ERK2
Dex
F
SGK1
4e-5 9e-6 2.7e-5
- Delta CT (HPRT1)
Control 1h Dex 1h Control 8h Dex 8h Control 24h Dex 24h
G Early repressed
FOXA1
0.358 0.001 0.002
I
GAB2
0.915 0.0051 0.019
IPTK1
0.56 0.0046 0.0015
RARA
0.87 0.126 0.0027
H
GREB1
0.56 0.94 0.032
TFF3
0.95 0.44 0.104
SLC7A5
0.86 0.358 0.014
SLC2A1
0.098 0.0031 0.0014

Figure EV3. GR activation decreases ER abundance and transcriptional activity.

(**A**) Immunoblots showing levels of GR, ER and ERK2 (loading control) in MCF-7 D538G cells treated with Dex for 72 h. (**B**) Immunoblots showing levels of ER, GR and ERK2 (loading control) in MCF-7 D538G cells treated with Dex (700 nM), Fulvestrant (1 μM), Camizestrant (1 μM) or Elacestrant (1 μM) for 72 h. (**C**, **D**) Immunoblots showing levels of GR, ER and ERK2 (loading control) in MCF-7 parental and T-47D cells treated with Dex for 7 days. (**E**) Immunoblots showing the levels of GR, ER and ERK2 (loading control) in MCF-7 D538G cells treated or not with Dex for the indicated times. (**F**) Scatter dot plots showing the mRNA levels of *SGK1* after GR activation for the indicated times, in MCF-7 D538G cells. $n = 3$ biological replicates. Two-tailed t-test. Data are presented as mean ± SD. (**G**) Scatter dot plots showing the mRNA levels of *FOXA1* after GR activation for the indicated times, in MCF-7 D538G cells. $n = 3$ biological replicates. Two-tailed *t* test. Data are presented as mean ± SD. (**H**) Scatter dot plots showing the mRNA level of *GREB1*, at indicated Dex treatment timepoints, in MCF-7 D538G cells. $n = 3$ biological replicates. Two-tailed *t* test. Data are presented as mean ± SD. (**I**) Scatter dot plots showing mRNA levels of E2-response genes that are late repressed in MCF-7 D538G cells after treatment with Dex or vehicle for the indicated times. $n = 3$ biological replicates. Two-tailed *t* test. Data are presented as mean ± SD.

 © The Author(s)

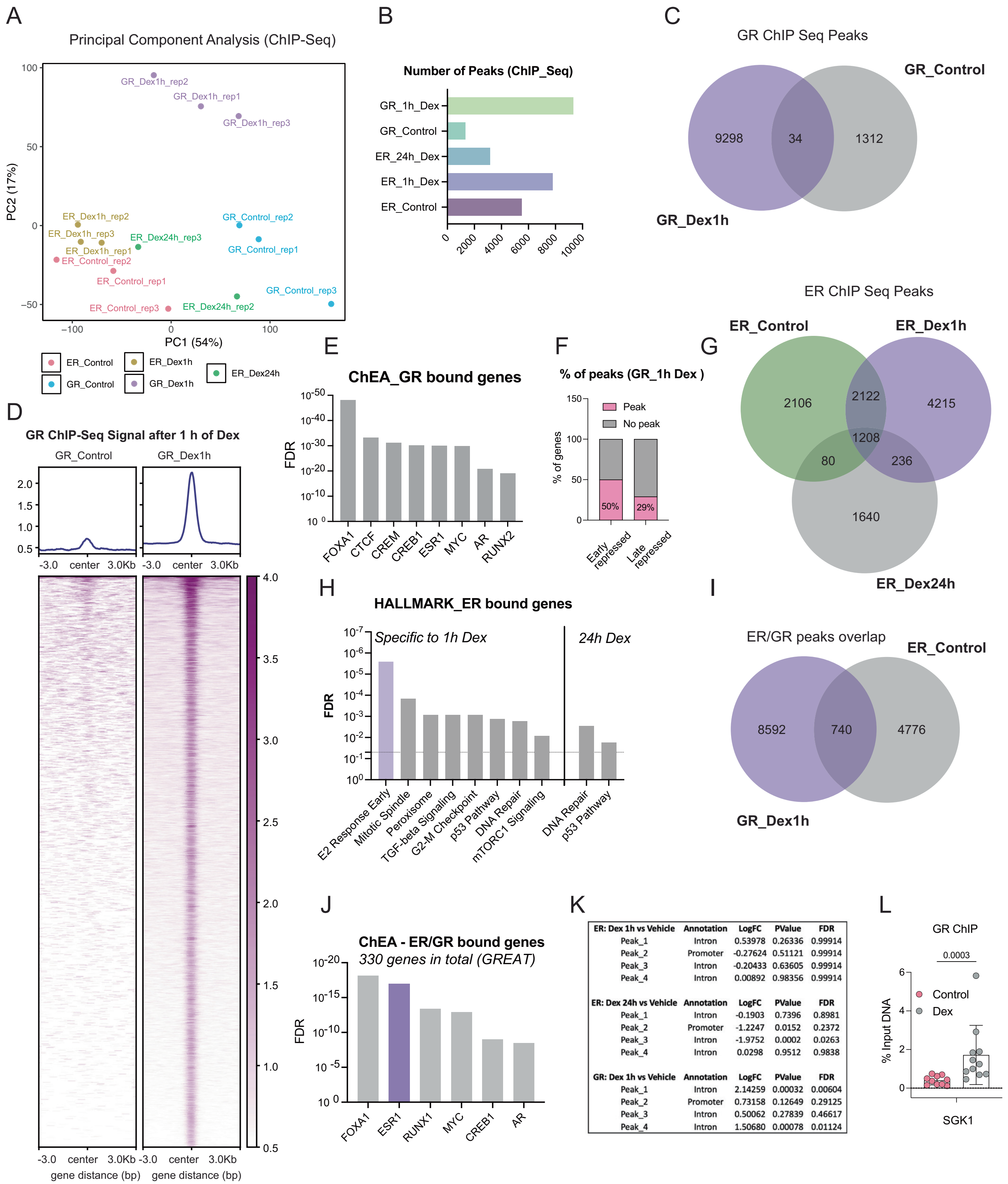

ER: Dex 1h vs Vehicle	Annotation	LogFC	PValue	FDR
Peak_1	Intron	0.53978	0.26336	0.99914
Peak_2	Promoter	-0.27624	0.51121	0.99914
Peak_3	Intron	-0.20433	0.63605	0.99914
Peak_4	Intron	0.00892	0.98356	0.99914
**ER: Dex 24h vs Vehicle**	**Annotation**	**LogFC**	**PValue**	**FDR**
Peak_1	Intron	-0.1903	0.7396	0.8981
Peak_2	Promoter	-1.2247	0.0152	0.2372
Peak_3	Intron	-1.9752	0.0002	0.0263
Peak_4	Intron	0.0298	0.9512	0.9838
**GR: Dex 1h vs Vehicle**	**Annotation**	**LogFC**	**PValue**	**FDR**
Peak_1	Intron	2.14259	0.00032	0.00604
Peak_2	Promoter	0.73158	0.12649	0.29125
Peak_3	Intron	0.50062	0.27839	0.46617
Peak_4	Intron	1.50680	0.00078	0.01124

© The Author(s)

Figure EV4. Analysis of ER and GR ChIP-Seq in MCF-7 *ESR1* D538G cells upon 1 h and 24 h Dex.

(**A**) Principal component analysis of the ChIP-Seq samples. One technical replicate of ER_Dex24h condition did not pass quality control and was removed due to low percentage (32.4%) of uniquely mapped aligned sequence in comparison to all other samples (> 80%). (**B**) Number of peaks detected per biological conditions in the ChIP-Seq experiment. (**C**) Venn diagram depicting the number of peaks and their overlap in the GR ChIP-Seq in absence or presence of Dex for 1 h. (**D**) Heatmap of GR ChIP-seq in absence or presence of Dex for 1 h in MCF-7 D538G cells. The heatmap is shown in a horizontal window of −/+ 3 kb around the center of the peaks. (**E**) ChEA transcription factor binding analysis of genes bound by GR in presence of Dex for 1 h. (**F**) Percentage of GR_1h Dex peaks identified by ChIP-Seq (in promoter, exonic, intronic and intergenic regions) located at the vicinity of early repressed and late repressed ER target genes, identified as downregulated by RNA-Seq upon 8 h and 24 h of Dex, respectively. (**G**) Venn diagram depicting the number of peaks and their overlap from the ER ChIP-Seq in absence or presence of Dex for 1 h or 24 h. (**H**) Hallmark functional annotation of genes bound by ER, specifically in the presence of Dex for 1 h or 24 h, using GREAT for peak to gene annotation. (**I**) Venn diagram depicting the number of peaks and their overlap between the ER_control and GR_Dex1h ChIP-Seq conditions. (**J**) ChEA transcription factor binding analysis of genes commonly bound by GR and ER, using GREAT for peak to gene annotation. (**K**) Table highlighting the Log FC, *P* value and FDR values corresponding to the 4 peaks (ER and/or GR) identified at the vicinity of the *ESR1* gene. (**L**) GR ChIP-qPCR of the GR target *SGK1* in MCF-7 D538G cells treated with Dex or vehicle for 1 h. Data are shown as percentage of input DNA. $n = 3$ experimental replicates. Two-tailed Mann–Whitney test. Data are presented as mean ± SD.

© The Author(s)

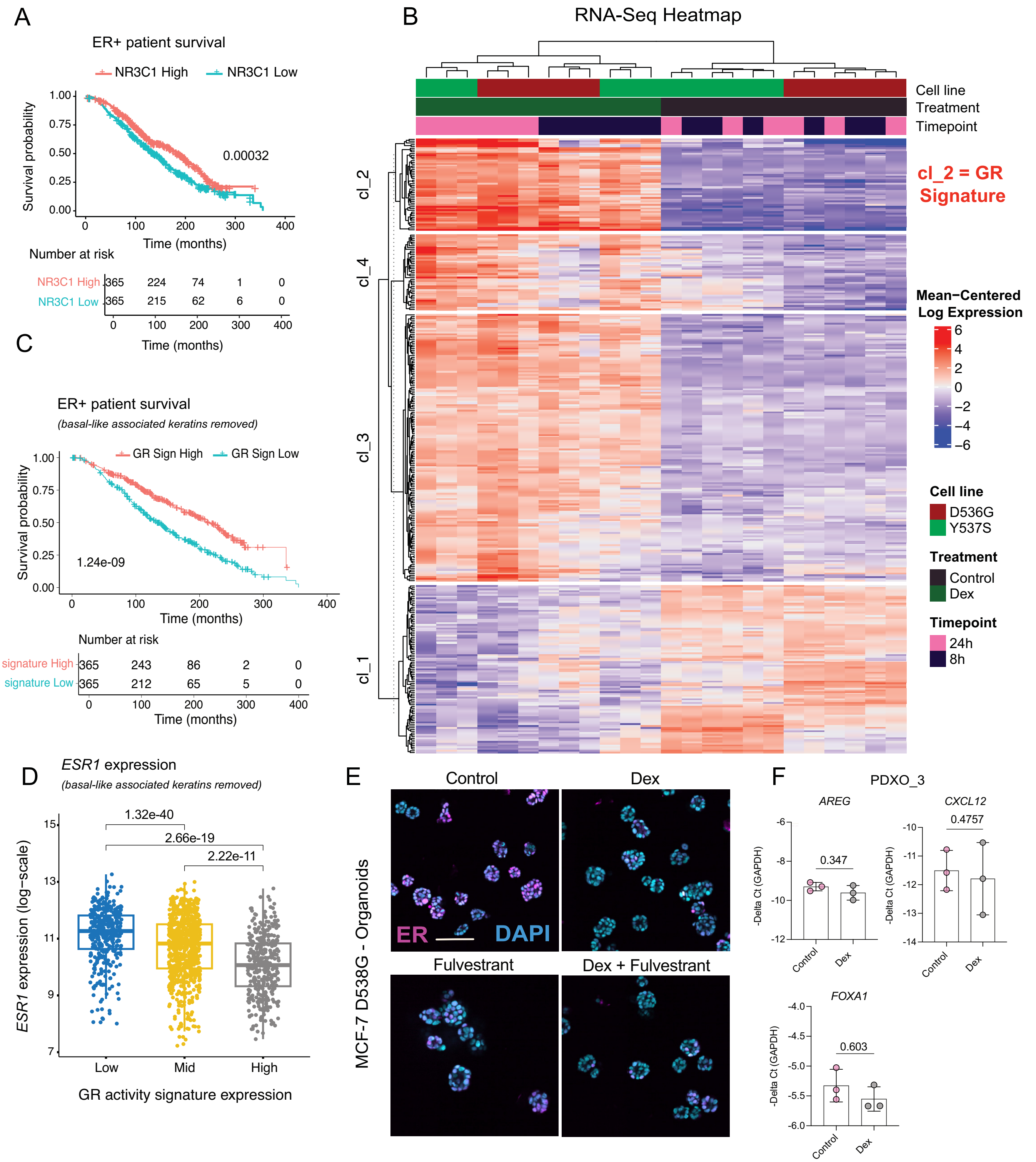
A
ER+ patient survival
NR3C1 High
NR3C1 Low
Survival probability
0.00032
Time (months)
Number at risk
NR3C1 High 365 224 74 1 0
NR3C1 Low 365 215 62 6 0
B
RNA-Seq Heatmap
Cell line
Treatment
Timepoint
cl_2
cl_4
cl_3
cl_1
cl_2 = GR Signature
Mean−Centered Log Expression
Cell line
D536G
Y537S
Treatment
Control
Dex
Timepoint
24h
8h
C
ER+ patient survival
(basal-like associated keratins removed)
GR Sign High
GR Sign Low
Survival probability
1.24e-09
Time (months)
Number at risk
signature High 365 243 86 2 0
signature Low 365 212 65 5 0
D
ESR1 expression
(basal-like associated keratins removed)
1.32e-40
2.66e-19
2.22e-11
ESR1 expression (log-scale)
Low
Mid
High
GR activity signature expression
E
Control
Dex
MCF-7 D538G - Organoids
ER
DAPI
Fulvestrant
Dex + Fulvestrant
F
PDXO_3
AREG
0.347
CXCL12
0.4757
FOXA1
0.603
-Delta Ct (GAPDH)
Control
Dex

Figure EV5. High *NR3C1* expression is associated with increased survival in patient with ER+ disease and GR activity signature generation.

(**A**) Kaplan–Meier survival plot of ER+ luminal patients (METABRIC annotation) showing that elevated GR expression is associated with prolonged survival in ER+ breast cancer patients. Patients were stratified based on high and low expression of *NR3C1*; Cox proportional hazard model with log-rank test. (**B**) Heatmap built from genes differentially regulated following RNA-Seq profiling of both MCF-7 D538G and Y537S models, treated or not with Dex for 8 h and 24 h. k-means (4) method was used for hierarchical clustering. Cluster_2 depicts the GR activity signature composed of 52 protein-coding genes, upregulated upon Dex treatment for 8 h and 24 h (RNA-Seq; Log FC > 2, FDR < 0.01), in both MCF-7 D538G and Y537S models. (**C**) Kaplan–Meier survival plot showing predictive value of GR activity signature (lacking basal-like keratins-encoding genes *KRT5, KRT6a, KRT6b, KRT6c* and *KRT16*) in patients with ER+ luminal breast cancer (METABRIC). Patients were stratified based on high and low GR activity signature score; Cox proportional hazard model with log-rank test. GR activity signature is composed of 52 protein-coding genes, upregulated upon Dex treatment for 8 h and 24 h in both MCF-7 Y537S and D538G models (see "Methods" for signature generation). (**D**) Graph showing *ESR1* mRNA expression in breast tumors from patients with ER+ disease (METABRIC), stratified according to the GR activity signature score (without basal-like associated keratins); low, intermediate, high. Wilcoxon matched pairs signed-rank test. (**E**) Representative confocal images (z-plan, 5 μM z-distance) of 3D-grown MCF-7 D538G cells after 3 days of the indicated treatments. Scale 50 μM. (**F**) Scatter dot plots showing the mRNA levels of *AREG, CXCL12* and *FOXA1* after 24 h of Dex treatment in PDXO_3 sample: $n = 3$ experimental replicates. Two-tailed *t* test. Data are presented as mean ± SD.

 © The Author(s)

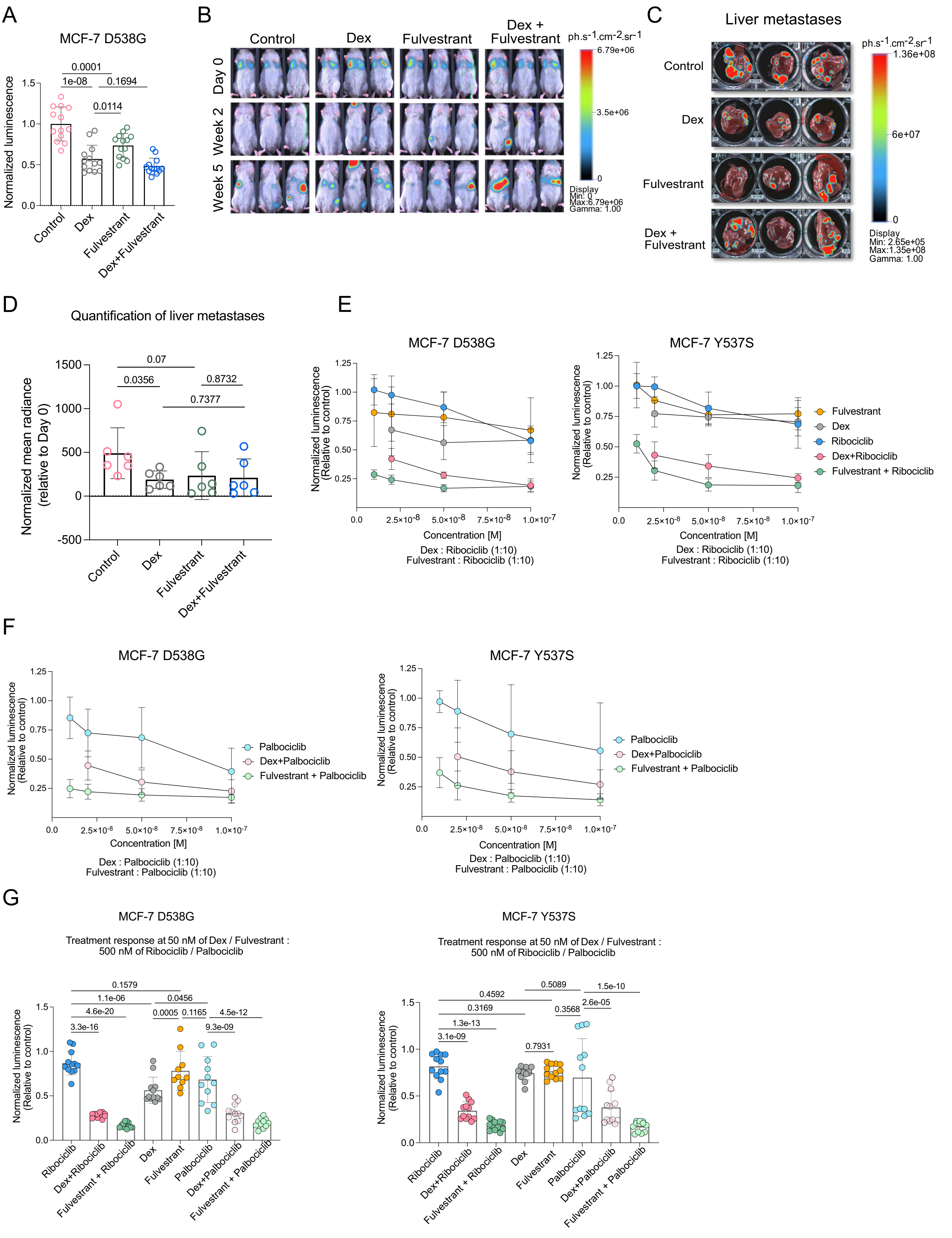
A
MCF-7 D538G
Normalized luminescence
0.0001
1e-08
0.1694
0.0114
Control
Dex
Fulvestrant
Dex+Fulvestrant
B
Control
Dex
Fulvestrant
Dex + Fulvestrant
Day 0
Week 2
Week 5
ph.s-1.cm-2.sr-1
6.79e+06
3.5e+06
Display
Min: 0
Max:6.79e+06
Gamma: 1.00
C
Liver metastases
Control
Dex
Fulvestrant
Dex + Fulvestrant
ph.s-1.cm-2.sr-1
1.36e+08
6e+07
Display
Min: 2.65e+05
Max:1.35e+08
Gamma: 1.00
D
Quantification of liver metastases
Normalized mean radiance (relative to Day 0)
0.07
0.0356
0.8732
0.7377
Control
Dex
Fulvestrant
Dex+Fulvestrant
E
MCF-7 D538G
MCF-7 Y537S
Normalized luminescence (Relative to control)
Concentration [M]
Dex : Ribociclib (1:10)
Fulvestrant : Ribociclib (1:10)
Fulvestrant
Dex
Ribociclib
Dex+Ribociclib
Fulvestrant + Ribociclib
F
MCF-7 D538G
MCF-7 Y537S
Normalized luminescence (Relative to control)
Concentration [M]
Dex : Palbociclib (1:10)
Fulvestrant : Palbociclib (1:10)
Palbociclib
Dex+Palbociclib
Fulvestrant + Palbociclib
G
MCF-7 D538G
Treatment response at 50 nM of Dex / Fulvestrant : 500 nM of Ribociclib / Palbociclib
0.1579
1.1e-06
0.0456
4.6e-20
0.0005
0.1165
4.5e-12
3.3e-16
9.3e-09
MCF-7 Y537S
Treatment response at 50 nM of Dex / Fulvestrant : 500 nM of Ribociclib / Palbociclib
0.5089
1.5e-10
0.4592
2.6e-05
0.3169
0.3568
1.3e-13
3.1e-09
0.7931
Normalized luminescence (Relative to control)
Ribociclib
Dex+Ribociclib
Fulvestrant + Ribociclib
Dex
Fulvestrant
Palbociclib
Dex+Palbociclib
Fulvestrant + Palbociclib

Figure EV6. GR activation decreases liver metastatic burden of MCF-7 *ESR1* mutant cancer cells compared to Fulvestrant, and synergizes with CDK4/6i in vitro.

(**A**) Bar graph showing the viability of MCF-7 D538G cells treated with vehicle, Dex, or Fulvestrant alone or in combination for 4 days; $n = 3$ experimental replicates, with 4–5 technical replicates each. Two-tailed Mann–Whitney test. Data are presented as mean ± SD. (**B**) Bioluminescence imaging of mice intravenously injected with MCF-7 D538G cells without prior treatments in vitro; $n = 6$ mice per group. Imaging was performed with Newton Vilber. (**C**) Representative bioluminescence images of liver metastases harvested upon sacrifice; $n = 6$ mice per group. (**D**) Bar graph showing the quantification of liver metastases harvested and measured at day 47; $n = 6$ mice per group. Two-tailed Mann–Whitney test. Data are presented as mean ± SD. (**E**) Graphs showing the viability of MCF-7 D538G and Y537S cells upon treatment with Dex, Fulvestrant, Ribociclib or the combination of drugs for 6 days. $n = 2$ experimental replicates, with 6 technical replicates each. Data are presented as mean ± SD. (**F**) Graphs showing the viability of MCF-7 D538G and Y537S cells upon treatment with Palbociclib or in combination with Dex or Fulvestrant for 6 days. $n = 2$ experimental replicates, with 6 technical replicates each. Data are presented as mean ± SD. (**G**) Scatter dot plots representing the viability of MCF-7 D538G and Y537S cells treated with 50 nM of Dex or Fulvestrant, 500 nM of Ribociclib or Palbociclib or the combinations as indicated. $n = 2$ experimental replicates, with 6 technical replicates each. One-way ANOVA test. Data are presented as mean ± SD.

 © The Author(s)
